# Cross-sectional and prospective relationships of endogenous progestogens and estrogens with glucose metabolism in men and women: a KORA F4/FF4 Study

**DOI:** 10.1136/bmjdrc-2020-001951

**Published:** 2021-02-11

**Authors:** Lina Hui Ying Lau, Jana Nano, Alexander Cecil, Florian Schederecker, Wolfgang Rathmann, Cornelia Prehn, Tanja Zeller, Andreas Lechner, Jerzy Adamski, Annette Peters, Barbara Thorand

**Affiliations:** 1 Institute of of Epidemiology, Helmholtz Zentrum München, German Research Center for Environmental Health, München-Neuherberg, Germany; 2 Institute for Medical Information Processing, Biometry, and Epidemiology (IBE), Ludwig-Maximilians-Universität (LMU), München, Germany; 3 International Helmholtz Research School for Diabetes, Helmholtz Zentrum München, German Research Center for Environmental Health, Neuherberg, Germany; 4 German Center for Diabetes Research (DZD), München-Neuherberg, Germany; 5 Research Unit, Molecular Endocrinology and Metabolism, Helmholtz Zentrum München, German Research Center for Environmental Health, Neuherberg, Germany; 6 Institute for Biometrics and Epidemiology, German Diabetes Center (DDZ), Leibniz Center for Diabetes Research at Heinrich Heine Universität, Düsseldorf, Germany; 7 Department of General and Interventional Cardiology, University Heart Center Hamburg, Hamburg, Germany; 8 German Center for Cardiovascular Research (DZHK), Partner Site Hamburg/Kiel/Lübeck, Lübeck, Germany; 9 Medizinische Klinik und Poliklinik IV, Ludwig-Maximilians-Universität (LMU), München, Germany; 10 Lehrstuhl für Experimentelle Genetik, Technische Universität München, München, Germany; 11 Department of Biochemistry, Yong Loo Lin School of Medicine, National University of Singapore, Singapore; 12 German Centre for Cardiovascular Research (DZHK), Partner Site Munich Heart Alliance, München, Germany

**Keywords:** progesterone, estrogens, diabetes mellitus, type 2

## Abstract

**Introduction:**

Relationships between endogenous female sex hormones and glycemic traits remain understudied, especially in men. We examined whether endogenous 17α-hydroxyprogesterone (17-OHP), progesterone, estradiol (E2), and free estradiol (fE2) were associated with glycemic traits and glycemic deterioration.

**Research design and methods:**

921 mainly middle-aged and elderly men and 390 perimenopausal/postmenopausal women from the German population-based Cooperative Health Research in the Region of Augsburg (KORA) F4/FF4 cohort study were followed up for a median of 6.4 years. Sex hormones were measured at baseline using mass spectrometry. We calculated regression coefficients (β) and ORs with 95% CIs using multivariable-adjusted linear and logistic regression models for Z-standardized hormones and glycemic traits or glycemic deterioration (ie, worsening of categorized glucose tolerance status), respectively.

**Results:**

In the cross-sectional analysis (n=1222 men and n=594 women), in men, 17-OHP was inversely associated with 2h-glucose (2hG) (β=−0.067, 95% CI −0.120 to −0.013) and fasting insulin (β=−0.074, 95% CI −0.118 to −0.030), and positively associated with Quantitative Insulin Sensitivity Check Index (QUICKI) (β=0.061, 95% CI 0.018 to 0.105). Progesterone was inversely associated with fasting insulin (β=−0.047, 95% CI −0.088 to −0.006) and positively associated with QUICKI (β=0.041, 95% CI 0.001 to 0.082). E2 was inversely associated with fasting insulin (β=−0.068, 95% CI −0.116 to −0.020) and positively associated with QUICKI (β=0.059, 95% CI 0.012 to 0.107). fE2 was positively associated with glycated hemoglobin (HbA_1c_) (β=0.079, 95% CI 0.027 to 0.132). In women, 17-OHP was positively associated with fasting glucose (FG) (β=0.068, 95% CI 0.014 to 0.123). fE2 was positively associated with FG (β=0.080, 95% CI 0.020 to 0.141) and HbA_1c_ (β=0.121, 95% CI 0.062 to 0.180). In the sensitivity analyses restricted to postmenopausal women, we observed a positive association between 17-OHP and glycemic deterioration (OR=1.518, 95% CI 1.033 to 2.264).

**Conclusions:**

Inter-relations exist between female sex hormones and glucose-related traits among perimenopausal/postmenopausal women and insulin-related traits among men. Endogenous progestogens and estrogens appear to be involved in glucose homeostasis not only in women but in men as well. Further well-powered studies assessing causal associations between endogenous female sex hormones and glycemic traits are warranted.

**Significance of this study**

**What is already known about this subject?**

- Endogenous progesterone and estradiol (E2) were associated with type 2 diabetes (T2D) and related glycemic traits in previous cross-sectional studies in postmenopausal women.

**What are the new findings?**

- We demonstrated that endogenous progesterone, 17α-hydroxyprogesterone (17-OHP), the product of progesterone hydrolysis, and E2 are independently associated with glycemic traits in men as well.
- Among postmenopausal women only, we demonstrated a positive association of endogenous 17-OHP with fasting glucose and glycemic deterioration.

**How might these results change the focus of research or clinical practice?**

- Although regarded as female sex hormones, endogenous progestogens and estrogens appear to be involved in glucose homeostasis not only in women but in men as well.

## Introduction

Extensive evidence from human and animal studies suggests that sex hormones are involved in modifying cardiometabolic risk, in particular diabetes development.^1^ These differences in risk may be explained by changes in body composition, alterations in glucose metabolism, and insulin sensitivity due to declining sex hormone concentrations associated with aging and menopause.^1^ However, whether glycemic traits specifically mediate the relationship between female sex hormones and glycemic deterioration remains controversial.^2 3^

Estrogens and progestogens comprise female sex hormones. Estradiol (E2) is the most potent and abundant endogenous estrogen. Higher levels of endogenous E2 have been associated with increased type 2 diabetes (T2D) risks in several population-based settings.^4 5^ Conversely, when used in hormone replacement therapy (HRT) E2 confers beneficial effects on glycemic control by reducing glycated hemoglobin (HbA_1c_) levels,^6^ fasting glucose (FG), and fasting insulin.^7^ Another endogenous hormone - progesterone, important especially during pregnancy, has been found to have positive associations with FG and HbA_1c_, and inverse associations with HOMA-β in both men and women.^8^ The product of progesterone hydrolysis, 17α-hydroxyprogesterone (17-OHP), has been observed to be elevated in patients with T2D.^9^ A study conducted in pregnant women showed that administration of 17-OHP caproate, a progestin-only contraceptive used to prevent preterm delivery, was associated with increased postchallenge glucose levels and increased risk of gestational diabetes (GD).^10^ Notably, women who develop GD are at higher risk of developing T2D later in life.^11^

Both estrogens and progestogens exist endogenously in men as well, but they are not considered as clinically relevant as they are in women^12^—leading to the lack of studies regarding these sex hormones in men.^5^ There is evidence concerning detrimental effects of estrogen deficiency in men.^13 14^ However, evidence for progestogens is limited.^8 15^ Available studies involving endogenous estrogen are mainly cross-sectional, have limited sample sizes, and lack comprehensive glycemic outcomes. Additionally, we are not aware of any epidemiological study to date investigating endogenous 17-OHP as an exposure.

Therefore, this study was conducted to explore the associations of endogenous 17-OHP, progesterone, E2, and free estradiol (fE2) with FG, 2h-glucose (2hG), HbA_1c_, fasting insulin, and Quantitative Insulin Sensitivity Check Index (QUICKI), separately in men and in perimenopausal/postmenopausal women. Furthermore, we examined prospective associations of these female sex hormones with glycemic deterioration defined as aberrant progressions from NGT or pre-diabetes to either pre-diabetes or diabetes during 6.4 years of follow-up.

## Methods

### Study population and selection criterions

**Figure 1:**
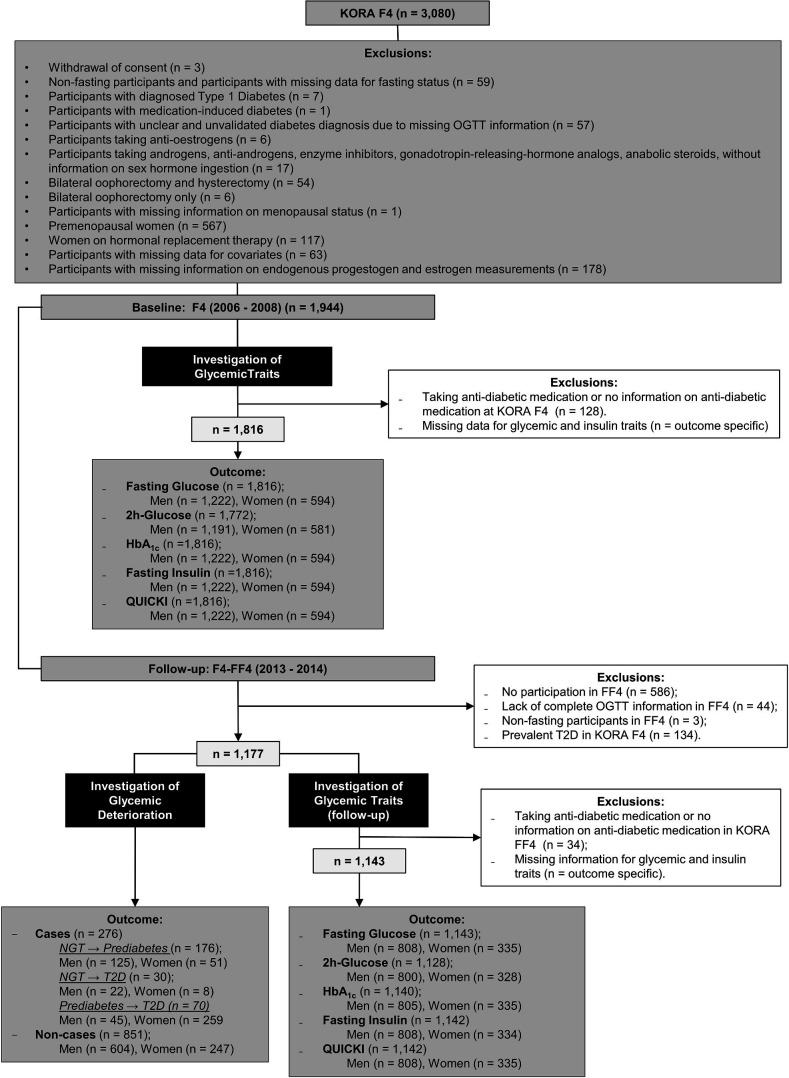
Flowchart showing sample sizes and exclusions. HbA_1c_, glycated hemoglobin; KORA, Cooperative Health Research in the Region of Augsburg; NGT, normoglycemia; OGTT, oral glucose tolerance test; QUICKI, Quantitative Insulin Sensitivity Check Index; T2D, type 2 diabetes.

The data for the study were obtained from the Cooperative Health Research in the Region of Augsburg (KORA) baseline (F4) (2006–2008) and follow-up (FF4) studies (2013–2014). Both studies are follow-up examinations of the KORA S4 study (1999–2001) conducted in Augsburg, Southern Germany, and two surrounding counties. The study design has been described previously in detail.^16^ The KORA F4 study included 3080 participants aged between 32 and 81 years, of whom 2161 also participated in KORA FF4. Three participants who withdrew consent were removed from the analyses. After further exclusions as described in figure 1, the final sample for the cross-sectional analysis comprised 1816 participants (1222 men and 594 women), while the prospective analysis sample comprised 1311 participants (921 men and 390 women). Participants taking antidiabetic medications were excluded from both cross-sectional and prospective analyses examining continuous glycemic traits as outcomes.

### Assessment of the outcomes

Previously known T2D was a self-report that could be validated by a physician or medical chart review, or as self-reported current use of glucose-lowering medication. Participants without known T2D were given a standard 75 g, oral glucose tolerance test (OGTT). Blood samples were taken without stasis after an overnight fast of ≥8 hours and 2 hours after glucose solution ingestion. Serum glucose was measured using hexokinase-G6PD (GLUFlex; Dade Behring, USA). In KORA FF4, glucose levels were quantified in serum either by using the glucose colorimetric assay (Dimension Vista 1500 System; Siemens Healthcare Diagnostics, USA) or the GLUC3 assay (Cobas c702; Roche Diagnostics GmbH, Germany). No calibration was needed for glucose as the double measurements were very similar. Normoglycemia (NGT) (ie, FG <6.1 mmol/L and 2hG <7.8 mmol/L), pre-diabetes (FG ≥6.1 mmol/L but <7.0 mmol/L, and 2hG <7.8 mmol/L (isolated impaired fasting glucose (IFG)) or FG of <6.1 mmol/L and 2hG ≥7.8 mmol/L but <11.1 mmol/L (isolated impaired glucose tolerance (IGT)), or both (IFG and IGT)), and newly-diagnosed diabetes (FG ≥7.0 mmol/L or 2hG ≥11.1 mmol/L) were defined according to the 1999/2006 WHO criteria.^17^ In KORA F4, HbA_1c_ was quantified in hemolysed whole blood using cation-exchange high-performance liquid chromatography (HPLC) (Adams HA 8160 Hemoglobin Analysis System; A. Menarini Diagnostics, Italy). In KORA FF4, HbA_1c_ concentrations were determined using ion-exchange HPLC (Variant II Turbo HbA_1c_ Kit; Bio-Rad Laboratories, USA). In KORA F4, fasting insulin was measured in thawed serum by an elctrochemiluminescence immunoassay (Cobas e602 Immunoassay Analyser; Roche Diagnostics GmbH, Germany). In KORA FF4, fasting insulin was quantified using either solid phase enzyme-labeled chemiluminescent immunometric assay (Immulite 2000 Systems Analyser, Siemens) or electrochemiluminescence immunoassay (Cobas e602 Immunoassay Analyser; Roche Diagnostics GmbH, Germany). Due to the change in measurement instruments and assays in KORA FF4, calibration was required for insulin measurements. This has been described previously in detail.^18^ QUICKI was used as a measure of insulin sensitivity and was calculated using the following formula: QUICKI=1/(log_10_(FG)+log_10_(fasting insulin)), with FG in milligram per decilitre and fasting insulin in microunit per millilitre. Glycemic deterioration was defined as the transition from NGT to pre-diabetes, NGT to T2D, and pre-diabetes to T2D from F4 to FF4. For this investigation, 135 participants with prevalent T2D at F4 were excluded, leading to a final sample for this analysis of 851 non-cases and 278 cases (online supplemental figure 1).

10.1136/bmjdrc-2020-001951.supp1 Supplementary data

### Assessment of the exposures: sex hormone measurements

Progesterone, 17-OHP, and E2 were quantified in serum using liquid chromatography–electrospray ionization–tandem mass spectrometry and the Absolute*IDQ* Stero17 Kit (BIOCRATES Life Sciences, Austria) (online supplemental material 1).^19^ The calibration, imputation, and normalization of sex hormone measurements are described in detail in online supplemental material 2. fE2 concentrations were estimated based on measured sex hormone-binding globulin (SHBG), E2, and albumin using the formula derived by Rinaldi *et al*^20^ (online supplemental material 3). SHBG in serum was quantified using the ARCHITECT SHBG assay, a chemiluminescent microparticle immunoassay (Abbott Laboratories, USA). Albumin in serum was quantified using immunonephelometry (ALB Flex; Dade Behring, Germany).

10.1136/bmjdrc-2020-001951.supp12 Supplementary data

10.1136/bmjdrc-2020-001951.supp13 Supplementary data

10.1136/bmjdrc-2020-001951.supp14 Supplementary data

### Assessment of covariates

In KORA F4, total cholesterol and high-density lipoprotein (HDL) cholesterol were measured in fresh serum by enzymatic methods (CHOL Flex and AHDL Flex, Dade Behring). Triglycerides were measured in fresh serum enzymatically (glycerine phosphate oxidase peroxidase method) (TGL Flex, Dade Behring). C reactive protein (CRP) was quantified from frozen plasma using a high-sensitivity latex-enhanced nephelometric assay (BN II Analyzer, Dade Behring). Thyroid-stimulating hormone (TSH) was measured using electrochemiluminescent methods (Dimension Vista Systems; Siemens, Germany). Serum creatinine was measured in fresh serum with a modified Jaffe test (KREA Flex, Dade Behring) according to IDMS standards. The estimated glomerular filtration rate (eGFR) was calculated using the Chronic Kidney Disease Epidemiology Collaboration (CKD-EPI) formula.^21^ Information on age, sex, statin medication, hypertension, smoking status, alcohol consumption, physical activity, and history of parental diabetes was assessed using a standardized interview, performed by trained medical staff. Hypertension was defined as having a blood pressure of >140/90 mm Hg or taking antihypertensive medication, given that the participants were aware of having hypertension. Information on medication use within 7 days before examination was obtained from a database.^22^ Smoking status was categorized as never smoked, former smokers, and current smokers (smoking≥1 cigarette a day). Alcohol consumption was categorized into three groups: no consumption (0 g/day), moderate consumption (men 0.1–29.9 g/day and women 0.1–19.9 g/day), and high consumption (men≥40 g/day and women≥20 g/day). Physical activity was estimated through two separate four-category interview questions regarding the time spent per week on sports activities in summer and winter. Possible answers were (1)>2 hours, (2) 1–2 hours, (3)<1 hour, and (4) none. Participants who had a total score of <5, obtained by summing the numbers (1)–(4) relating to winter and summer, were classified to be ‘physically active’.^23^ Parental diabetes was categorized as no parental diabetes history, unknown parental diabetes history, or ≥1 parent with diabetes history.

### Statistical analyses

Baseline characteristics of normally distributed continuous covariates are expressed as means with corresponding SD. Non-normally distributed continuous covariates were expressed as medians with the corresponding 25th and 75th percentiles. Proportions are expressed as percentages. Differences between participants with and without glycemic deterioration were calculated using Mann-Whitney U tests, while differences in categorical variables were compared using Kruskal-Wallis tests. Skewed variables were natural log (ln)-transformed to improve normalization. Z-standardization was performed sex-specifically for exposures, respectively, to achieve comparability despite their different scales. Due to significant interactions between sex and some hormones regarding glycemic traits (online supplemental table 1), sex-stratified analyses were employed throughout this study.

10.1136/bmjdrc-2020-001951.supp4 Supplementary data

Linear regression was performed to explore the cross-sectional and prospective relationships between progestogens and estrogens with glycemic traits, such as FG, 2hG, HbA_1c_, fasting insulin, and QUICKI. β-estimates with 95% CIs for Z-scores of sex hormones are given as per one sex-specific SD increase in ln-transformed progestogens and estrogens, respectively. Association analyses focusing on pathophysiological mechanisms were adjusted for F4 T2D risk factors such as age, waist circumference, height, ln(triglycerides), total cholesterol:HDL cholesterol ratio, actual hypertension (yes/no), and use of statins (yes/no) (model 1). Additional adjustments included lifestyle risk factors such as smoking status (never/former/current), alcohol consumption (no/low/high), and physical activity (active/inactive), and additionally, ln(CRP) (continuous), ln(TSH) (continuous), eGFR (continuous), and history of parental diabetes (no history/unknown history/≥1 parent with diabetes) (model 2). In the prospective analyses, there were further adjustments for F4 values of respective glycemic traits.

We calculated ORs with 95% CIs using logistic regression to investigate associations between female sex hormones and glycemic deterioration. These associations were additionally investigated for non-linearity by testing whether the introduction of a restricted cubic spline, with three knots placed at the 30th, 60th, and 90th percentiles, would improve the model fit where medians were set as the reference values for each exposure.

The confounders that constitute our models are common T2D risk factors, along with variables that affect T2D pathophysiology and circulating sex hormone levels. We adjusted for statin usage as they can increase T2D risks.^24^ TSH was adjusted due to its impact on sex hormone metabolism.^25^ We performed several sensitivity analyses: (1) further adjusting models containing E2 as the exposure for SHBG as SHBG determines circulating fE2 levels,^26^ (2) further adjusting models with progesterone as the exposure for albumin as it binds extensively to albumin,^27^ (3) excluding perimenopausal women (n=66) as sex hormone fluctuates during perimenopause. Given the homogeneity of progestogens, interaction analyses between 17-OHP and progesterone were performed where significant associations were present to determine whether combinations of different progestogen concentrations would influence the outcomes. The interaction effects are presented using contour plots. Significance levels were based on two-sided tests, where p values of ≤0.05 were considered statistically significant. Statistical analyses were performed using R V.3.6.1.

## Results

### Baseline characteristics

Men and women with glycemic deterioration (ie, cases) were older; had larger waist circumference and higher triglyceride levels and total cholesterol:HDL cholesterol ratio; were more likely to be hypertensive; had elevated CRP; and were more likely to have ≥1 parent with diabetes compared with those without glycemic deterioration (ie, non-cases). Among women, cases had lower TSH levels. In men, cases had higher 17-OHP, E2, and fE2 levels compared with non-cases. In women, sex hormone levels were not significantly different between cases and non-cases. At F4 and FF4, cases had higher FG, 2hG, HbA_1c_, fasting insulin, and lower QUICKI values compared with non-cases in men and women (table 1).

**Table 1:** Descriptive characteristics of men and perimenopausal/postmenopausal women in KORA F4/FF4*

	Men (n=796)			Perimenopausal/postmenopausal women† (n=331)		
	Non-cases‡ (n=604)	Cases‡ (n=192)	P value	Non-cases‡ (n=247)	Cases‡ (n=84)	P value
Age (years)	51.6 (12.2)	58.5 (10.9)	<0.001	59.7 (8.5)	62.4 (8.5)	0.005
Height (cm)	177 (6.9)	175 (6.9)	<0.001	161 (6.1)	160 (6.4)	0.068
Waist circumference (cm)	95 (89, 103)	101 (95, 109)	<0.001	85 (78, 93)	93 (88, 102)	<0.001
Triglycerides (mmol/L)	1.26 (0.87, 1.74)	1.56 (1.07, 2.4)	<0.001	1.03 (0.75, 1.39)	1.24 (0.99, 1.78)	<0.001
Total cholesterol/HDL cholesterol	4.17 (3.51, 5.00)	4.54 (3.85, 5.47)	<0.001	3.60 (3.02, 4.19)	4.10 (3.47, 4.78)	<0.001
Hypertension (%)	28.6	50.0	<0.001	28.3	52.3	<0.001
Statin use (%)	6.9	17.7	<0.001	10.5	9.5	0.957
Smoking status						
Never (%)	32.9	37.5	0.081	54.3	63.1	0.306
Former (%)	46.4	49.0		31.2	27.4	
Current (%)	20.7	13.5		14.6	9.5	
Alcohol consumption						
None (%)	16.4	20.3	0.435	35.2	45.2	0.258
Moderate (%)	66.2	64.1		49.8	42.9	
High (%)	17.4	15.6		14.9	11.9	
Physically active (%)	59.6	57.3	0.629	63.9	51.2	0.052
CRP (mg/L)	0.85 (0.44, 1.78)	1.28 (0.67, 2.28)	<0.001	1.05 (0.53, 2.05)	1.96 (1.02, 4.06)	<0.001
eGFR (mL/min/1.73 m²)	92.1 (14.9)	86.3 (13.3)	<0.001	86.2 (14.5)	84.5 (14.7)	0.354
TSH (mIU/L)	1.25 (0.87, 1.85)	1.36 (0.91, 1.99)	0.085	1.32 (0.87, 1.88)	1.18 (0.75, 1.65)	0.038
Parental history of diabetes (%)						
Both parents without diabetes	65.9	48.9	<0.001	60.3	47.6	0.091
Unknown parental history	14.1	26.6		16.2	17.8	
≥1 parent with diabetes	20.0	24.5		23.5	34.5	
17-OHP (nmol/L)	2.88 (2.12,3.94)	2.51 (1.93, 3.52)	0.002	0.77 (0.52, 1.21)	0.87 (0.52, 1.34)	0.267
Progesterone (nmol/L)	0.20 (0.12, 0.32)	0.17 (0.10, 0.31)	0.063	0.12 (0.04, 0.23)	0.12 (0.06, 0.19)	0.762
E2 (nmol/L)	0.49 (0.36, 0.68)	0.42 (0.29, 0.54)	<0.001	0.17 (0.09, 0.28)	0.17 (0.10, 0.27)	0.970
fE2 (nmol/L)	0.010 (0.007, 0.016)	0.008 (0.006, 0.013)	<0.001	0.003 (0.001, 0.004)	0.003 (0.001,.005)	0.406
F4						
FG (mmol/L)	5.22 (4.94, 5.50)	5.61 (5.33,5.89)	<0.001	5.00 (4.78, 5.33)	5.42 (5.17,5.94)	<0.001
2hG (mmol/L)	5.50 (4.67, 6.33)	6.61 (5.71, 7.40)	<0.001	5.39 (4.56, 6.47)	6.72 (5.77, 7.40)	<0.001
HbA_1c_ (%)	5.3 (5.1, 5.5)	5.5 (5.4, 5.8)	<0.001	5.4 (5.3, 5.6)	5.6 (5.4, 5.9)	<0.001
Fasting insulin (pmol/L)	50.0 (36.0, 66.0)	66.0 (49.0, 102.0)	<0.001	46.2 (35.4,66.0)	66.0 (47.3, 90.0)	<0.001
QUICKI	0.35 (0.028)	0.33 (0.028)	<0.001	0.35 (0.023)	0.33 (0.026)	<0.001
FF4						
FG (mmol/L)	5.44 (5.16, 5.72)	6.22 (5.77, 6.55)	<0.001	5.27 (4.94, 5.61)	6.05 (5.55, 6.38)	<0.001
2hG (mmol/L)	5.52 (4.66, 6.50)	8.38 (7.33, 9.99)	<0.001	5.66 (4.72,6.49)	8.27 (7.77,10.1)	<0.001
HbA_1c_ (%)	5.4 (5.1, 5.5)	5.6 (5.4, 6.0)	<0.001	5.4 (5.3,5.6)	5.7 (5.4, 5.9)	<0.001
Fasting insulin (pmol/L)	49.9 (36.6, 74.9)	81.0 (56.5, 117.6)	<0.001	52.2 (36.5, 74.9)	84.0 (58.6,105.3)	<0.001
QUICKI	0.34 (0.029)	0.32 (0.029)	<0.001	0.35 (0.029)	0.32 (0.024)	<0.001

*Men and perimenopausal/postmenopausal women not taking antidiabetic medication.

†Perimenopausal/postmenopausal women not on oral contraceptives or HRT.

‡Comparison of descriptive characteristics of the study population with (case) and without (non-case) glycemic deterioration. Glycemic deterioration (yes/no) is defined as the progression from NGT to pre-diabetes, NGT to T2D, and pre-diabetes to T2D from F4 to FF4.

CRP, C reactive protein; E2, Estradiol; eGFR, Estimated glomerular filtration rate (creatinine-based); F4, baseline; fE2, Free estradiol; FF4, follow-up; FG, Fasting glucose; HbA_1c_, Glycated hemoglobin; HDL, High-density lipoprotein; 2hG, 2h-glucose; HRT, Hormone replacement therapy; KORA, Cooperative Health Research in the Region of Augsburg; NGT, Normoglycemia; 17-OHP, 17α-hydroxyprogesterone; QUICKI, Quantitative Insulin Sensitivity Check Index; T2D, type 2 diabetes; TSH, Thyroid-stimulating hormone.

### Cross-sectional associations of endogenous progestogens and estrogens with glycemic traits

**Figure 2:**
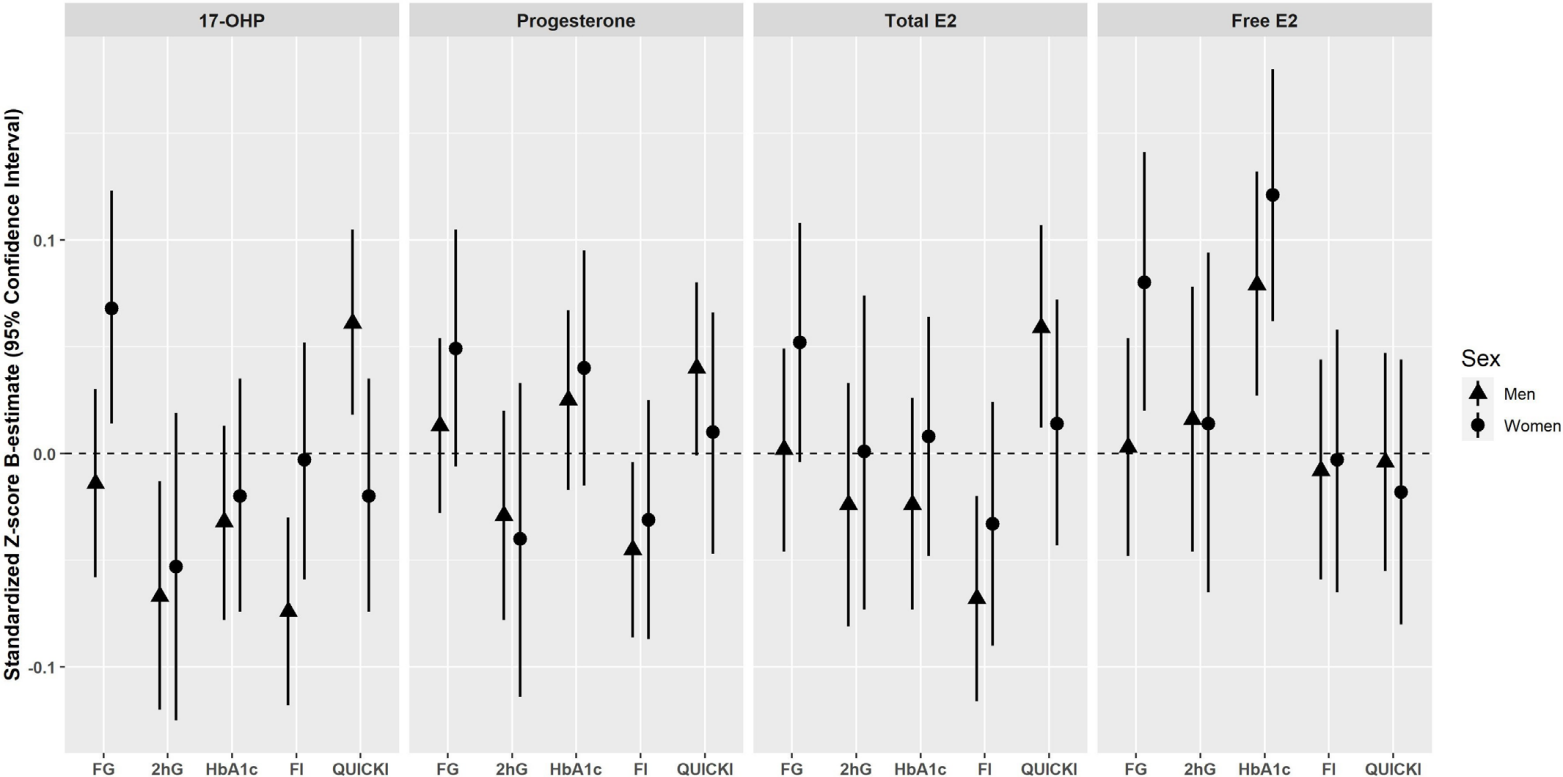
Cross-sectional associations of endogenous progestogens and estrogens with glycemic traits in men and women of the KORA F4 cohort*. Results are expressed as the change in 1 log unit of the continuous outcome (standardized Z-score β-estimate with 95% CI) per 1 sex-specific SD increase in the respective progestogens and estrogens adjusted for baseline age, waist circumference, height, triglycerides, total cholesterol:high-density lipoprotein cholesterol ratio, hypertension, statin use, smoking status, alcohol consumption, physical activity, CRP, eGFR, TSH, and parental history of diabetes (model 2). *Men and perimenopausal/postmenopausal women who did not take antidiabetic medication. CRP, C reactive protein; E2, Estradiol; eGFR, estimated glomerular filtration rate; F4, baseline; FG, fasting glucose; FI, fasting insulin; HbA_1c_, glycated hemoglobin; 2hG, 2h-glucose; KORA, Cooperative Health Research in the Region of Augsburg; 17-OHP, 17α-hydroxyprogesterone; QUICKI, Quantitative Insulin Sensitivity Check Index; TSH, thyroid-stimulating hormone.

Cross-sectional associations are summarized in figure 2. In men, 17-OHP was inversely associated with 2hG (β=−0.074, 95% CI −0.130 to −0.019), fasting insulin (β=−0.093, 95% CI −0.140 to −0.046), and positively associated with QUICKI (β=0.079, 95% CI 0.032 to 0.126) after adjustment using model 1. On further adjustment (model 2), the significance persisted for all three outcomes: 2hG (β=−0.067, 95% CI −0.120 to −0.013), fasting insulin (β=−0.074, 95% CI −0.118 to −0.030), and QUICKI (β=0.061, 95% CI 0.018 to 0.105). Inverse associations were detected between progesterone and fasting insulin in model 1 (β=−0.052, 95% CI −0.096 to −0.008). The association remained significant after further adjustment for T2D risk factors (model 2: β=−0.045, 95% CI −0.086 to −0.004) and additional adjustment for albumin (β=−0.047, 95% CI −0.088 to −0.006). Also, progesterone was initially associated with QUICKI in model 1 (β=0.045, 95% CI 0.001 to 0.088), but the association became non-significant after further adjustment (model 2, β=0.040, 95% CI −0.001 to 0.080) (online supplemental table 2). In women, 17-OHP was positively associated with fasting glucose (β=0.071, 95% CI 0.015 to 0.127) in model 1. The significance persisted after further adjustment in model 2 (β=0.068, 95% CI 0.014 to 0.123). No further associations were found between 17-OHP and progesterone and glycemic traits in women (online supplemental table 3).

10.1136/bmjdrc-2020-001951.supp5 Supplementary data

10.1136/bmjdrc-2020-001951.supp6 Supplementary data

In men, after adjustment using model 1, E2 was inversely associated with 2hG (β=−0.059, 95% CI −0.118 to −0.001), fasting insulin (β=−0.113, 95% CI −0.163 to −0.062), and positively associated with QUICKI (β=0.105, 95% CI 0.054 to 0.155). After further adjustment in model 2, significant associations ceased for 2hG (β=−0.024, 95% CI −0.081 to 0.033), while it persisted for fasting insulin (β=−0.068, 95% CI −0.116 to −0.020) and QUICKI (β=0.059, 95% CI 0.012 to 0.107). On further adjustment with SHBG, the associations of E2 with 2hG (β=−0.013, 95% CI −0.073 to 0.046) and fasting insulin (β=−0.055, 95% CI −0.105 to −0.005) did not change significantly. However, the association between E2 and QUICKI ceased (β=0.044, 95% CI −0.005 to 0.093). fE2 was found to be positively associated with HbA_1c_ after adjustment in models 1 (β=0.012, 95% CI 0.004 to 0.021) and 2 (β=0.079, 95% CI 0.027 to 0.132). No further associations were found between fE2 and glycemic traits in men. In women, no significant associations were observed between E2 and glycemic traits after adjustment in models 1 and 2, and after further adjustment for SHBG. However, fE2 was positively associated with fasting glucose after adjusting with models 1 and 2, respectively (model 2: β=0.080, 95% CI 0.020 to 0.141) and HbA_1c_ (model 2: β=0.121, 95% CI 0.062 to 0.180) (online supplemental table 3). Substitution of waist circumference and height with body mass index did not significantly change the results (data not shown).

In the sensitivity analyses, among men, the inverse association between progesterone and fasting insulin remained significant in model 2 after additional adjustment for albumin. As for the association between progesterone and QUICKI, additional adjustment for albumin in model 2 reinstated the significance (β=0.041, 95% CI 0.001 to 0.082), which was previously made insignificant after adjustment in model 2 (β=0.040, 95% CI −0.001 to 0.082) (online supplemental table 2). The positive association between E2 and QUICKI remained significant after additional adjustment with SHBG in model 2. In women, additional adjustments with albumin and SHBG did not significantly change the results (online supplemental table 3). After perimenopausal women were excluded, associations between sex hormones and fasting glucose, as well as HbA_1c_, generally became stronger. Specifically, progesterone (β=0.071, 95% CI 0.007 to 0.136) and E2 (β=0.076, 95% CI 0.014 to 0.137) became significantly associated with fasting glucose and progesterone with HbA_1c_ (β=0.071, 95% CI 0.008 to 0.133) (online supplemental table 3).

**Figure 3:**
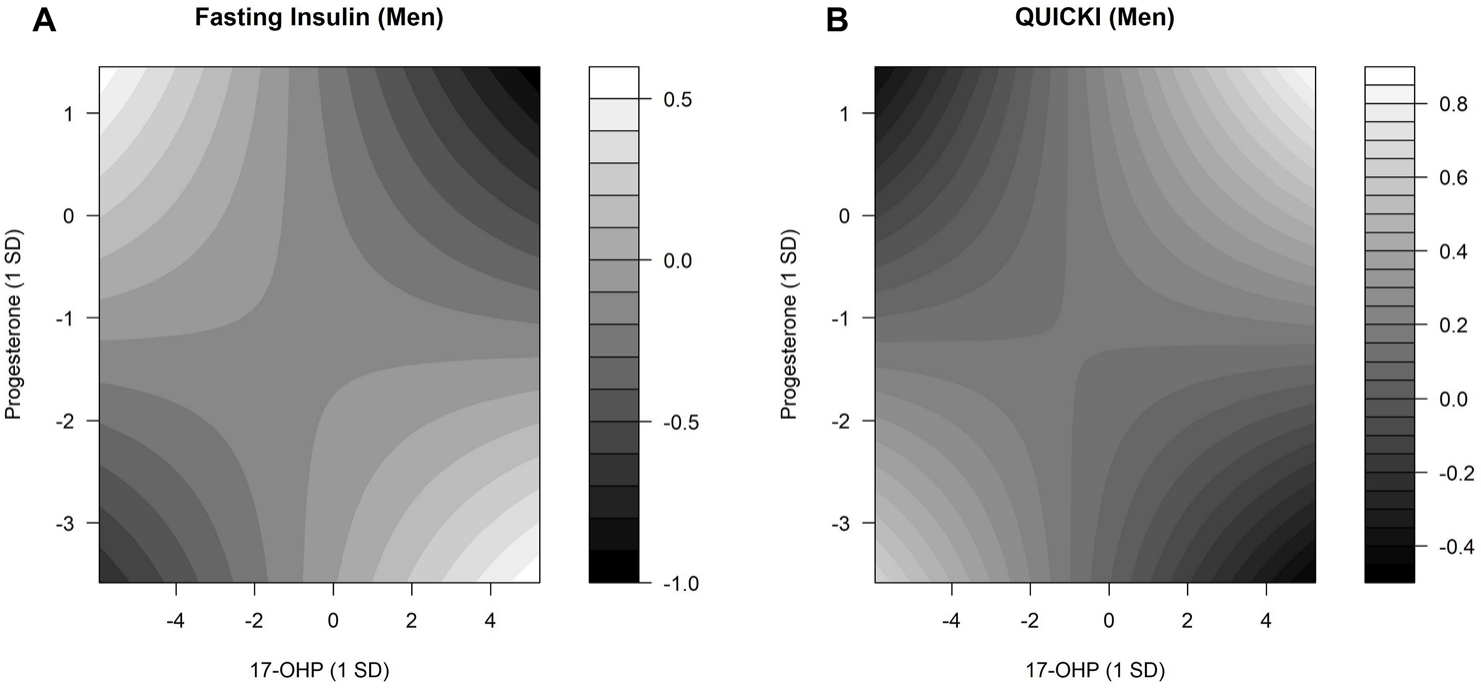
Interaction effects between 17-OHP and progesterone regarding fasting serum insulin and QUICKI. Contour plots estimated by linear regression models demonstrate the changes in fasting insulin and QUICKI for different concentrations of 17-OHP and progesterone. The predicted fasting serum insulin and QUICKI values were presented with gradients, ranging from black (low fasting insulin and QUICKI values) to white (high fasting insulin and QUICKI values). (A) P value for interaction=0.002. (B) P value for interaction=0.011. Linear predictions were adjusted for baseline age, waist circumference, height, triglycerides, total cholesterol:high-density lipoprotein cholesterol ratio, hypertension, statin use, smoking status, alcohol consumption, physical activity, CRP, eGFR, TSH, and parental diabetes history. 17-OHP, 17α-hydroxyprogesterone; CRP, C reactive protein; eGFR, estimated glomerular filtration rate; QUICKI, Quantitative Insulin Sensitivity Check Index; TSH, thyroid-stimulating hormone.

In men, there were interactions between 17-OHP and progesterone (online supplemental table 6). Selected results are shown in figure 3. Lower fasting insulin levels were observed when both 17-OHP and progesterone levels were at the lowest or highest (figure 3A). Higher QUICKI values were observed in men when both 17-OHP and progesterone concentrations were at the lowest or highest. Lower QUICKI values were observed in men with the highest progesterone and lowest 17-OHP levels and also with the highest 17-OHP and lowest progesterone levels (figure 3B). In women, no interactions were detected between 17-OHP and progesterone on fasting glucose (online supplemental table 6).

10.1136/bmjdrc-2020-001951.supp9 Supplementary data

### Glycemic deterioration

**Figure 4:**
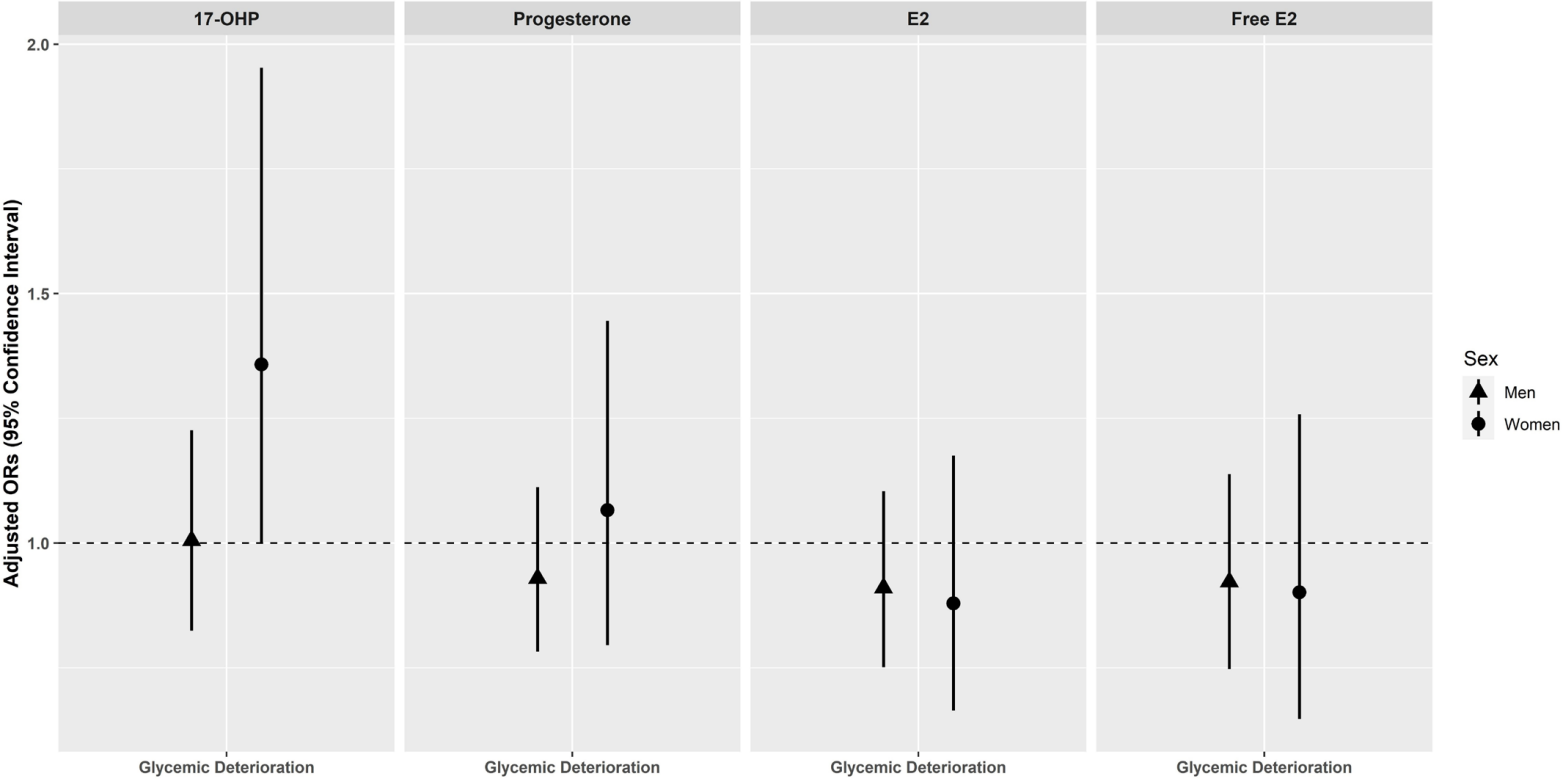
Association of endogenous progestogens and estrogens with glycemic deterioration in men and women of the KORA F4/FF4 cohort*. Adjusted ORs with 95% CIs for glycemic deterioration per 1 sex-specific SD increase in log-transformed progestogen and estrogen. ORs are adjusted for baseline age, waist circumference, height, triglycerides, total cholesterol:high-density lipoprotein cholesterol ratio, hypertension, statin use, smoking status, alcohol consumption, physical activity, CRP, eGFR (creatinine-based), TSH, and parental history of diabetes (model 2). *Men and perimenopausal/postmenopausal women without prevalent type 2 diabetes at baseline. CRP, C reactive protein; E2, Estradiol; eGFR, estimated glomerular filtration rate; F4, baseline; FF4, follow-up; KORA, Cooperative Health Research in the Region of Augsburg; 17-OHP, 17α-hydroxyprogesterone; TSH, thyroid-stimulating hormone.

No significant associations between progestogens and estrogens with glycemic deterioration were observed in men and women (figure 4). After removal of perimenopausal women in the sensitivity analysis, 17-OHP was significantly associated with glycemic deterioration in postmenopausal women (OR=1.518, 95% CI 1.033 to 2.264)) (online supplemental table 4). We also assessed for non-linear relationships across different progestogen and estrogen concentrations (online supplemental figure 2). However, there were no indications for significant non-linear relationships (online supplemental table 5).

10.1136/bmjdrc-2020-001951.supp7 Supplementary data

10.1136/bmjdrc-2020-001951.supp2 Supplementary data

10.1136/bmjdrc-2020-001951.supp8 Supplementary data

### Prospective associations of endogenous progestogens and estrogens with glycemic traits

In men, progesterone was positively associated with fasting insulin (β=0.052, 95% CI 0.005 to 0.098) and inversely associated with QUICKI (β=−0.048, 95% CI −0.095 to −0.000) after adjustment in model 1. However, associations between progesterone and fasting insulin (β=0.044, 95% CI −0.002 to 0.091) and QUICKI (β=−0.040, 95% CI −0.088 to 0.007) ceased after adjustment in model 2 (online supplemental table 7). In women, no associations were found between progestogens and estrogens and glycemic traits regardless of adjustments in models 1 and 2 and further adjustments for SHBG and albumin (online supplemental table 8).

10.1136/bmjdrc-2020-001951.supp10 Supplementary data

10.1136/bmjdrc-2020-001951.supp11 Supplementary data

## Discussion

In this population-based study of mainly middle-aged and elderly participants, we found that progestogens and estrogens were associated with glucose and insulin traits in men, whereas in women, associations were found only with glucose traits. Specifically, in the cross-sectional analyses in men, we found that higher levels of 17-OHP, progesterone, and E2 were associated with lower fasting insulin, whereas higher 17-OHP and E2 were associated with higher QUICKI values. Concerning glucose traits among men, higher 17-OHP levels were associated with lower 2hG concentrations whereas higher fE2 levels were associated with higher HbA_1c_ concentrations. Among women, positive associations were observed between 17-OHP and fasting glucose and between fE2 and fasting glucose as well as HbA_1c_. After exclusion of perimenopausal women, we observed significant associations of progesterone, 17-OHP and E2 with fasting glucose and of progesterone with HbA_1c_. Furthermore, we found significant interactions between 17-OHP and progesterone on fasting insulin levels and QUICKI in men. In the prospective analyses, we found no associations in both men and women after multivariable adjustment in the main analyses. However, in the sensitivity analysis, the exclusion of perimenopausal women revealed that postmenopausal women with elevated baseline 17-OHP levels had an increased risk of glycemic deterioration.

Congruent to our results, a cross-sectional study conducted in a rural Chinese population found positive associations of progesterone with fasting glucose, HbA_1c_, and an increased risk of prevalent pre-diabetes and T2D in men and women.^8^ Furthermore, in the study of Jiang *et al*^8^ in men and women, progesterone was inversely associated with HOMA-2β, an index of β-cell function, but not with fasting insulin as seen among men in the present study. The slightly diverging observations could be due to differences in ethnicity, lifestyle factors, socioeconomic status, and sample size between the populations. A recent study in men and women by Lu *et al*^9^ reported positive correlations between 17-OHP and fasting glucose, 2hG, and HbA_1c_. This was consistent with our observations of a positive association between fasting glucose and 17-OHP among women. However, the study by Lu *et al*^9^ performed correlation analyses without appropriate confounder adjustments, therefore limiting its interpretability. A Swedish longitudinal study (n=240) conducted among opposite-sex twins found no association between progesterone and diabetes risk.^15^ This corresponds to our null findings regarding the association of progestogens with glycemic deterioration. In the present study, the cross-sectional and prospective effect estimates of progesterone on fasting insulin and QUICKI show a change of direction in men. This could be due to the presence of (negative) confounding or random chance (given the insignificant results of model 2). However, our cross-sectional results are in line with current experimental evidence as described further.

Mechanisms by which progestogens alter glucose and insulin metabolism are nebulous, but there are some possible explanations. Elevated 17-OHP can induce hyperglycemia in female mice, and CYP17A1 is suggested to play a role in modulating this effect.^9^ CYP17A1 converts progesterone to 17-OHP,^28^ and Lu *et al*^9^ proposed that increased 17-OHP levels due to aberrant expression of CYP17A1 in obese mice increase blood glucose via the glucocorticoid (GC) receptor. GCs can confer hyperglycemia and gluconeogenesis^29^ and could explain the positive association between 17-OHP and fasting glucose in women. However, in men, we saw that 17-OHP levels were negatively associated with 2hG levels. Among men, higher 17-OHP levels could improve insulin sensitivity, thus lowering glucose levels. Specific variants in genes coding for CYP17A1 were suggestive of T2D susceptibility. Wang *et al*^30^ showed that polymorphism rs12413409, corresponding to CYP17A1 under-expression, was associated with increased fasting glucose only in men. Hence, the role of the polymorphism in glucose metabolism specific to men could explain our observations. We also observed interactions between 17-OHP and progesterone on fasting insulin in men. Imbalanced progestogen concentrations can cause aberrant GC receptor signaling due to competitive binding^31^ and may thereby contribute to suboptimal insulin levels. Consequently, perturbations in glucose homeostasis may arise. Until now, 17-OHP and diabetes risk have been implicated only in pregnant women.^10^ However, we showed that increased endogenous 17-OHP could also impact glucose homeostasis later in life among postmenopausal women. Fluctuating sex hormones during the cycle in perimenopausal women^32^ could have confounded our results when perimenopausal and postmenopausal women were analyzed together.

In men, E2 was negatively associated with fasting insulin levels and positively with insulin sensitivity in our study. Our observations are consistent with a study by Yan *et al,*^33^ where they found that treatment with E2 improves insulin sensitivity in hepatocytes. A Mendelian randomization study by Wang *et al*^34^ found a causative protective role of SHBG against T2D. However, weaker causal estimates of the causative protective role of SHBG compared with those observed from meta-analyses of prospective studies suggest that the observed protective role of SHBG could be confounded, as opposed to direct SHBG action. This is consistent with our results as we saw that the positive associations between E2 and insulin sensitivity were independent of SHBG and typical T2D risk factors. Our results showed persistent positive associations between fE2 and HbA_1c_ in both men and women. fE2 is the portion of E2 that is not bound to SHBG and is free to activate estrogen receptors (ERs). Under normal circumstances, E2 suppresses hepatic gluconeogenesis, potentially mediated through the activation of ERα-phosphoinositide 3-kinase-Akt-Foxo1 signaling.^33^ Due to the age-related E2 decline in both men and postmenopausal women, we hypothesize that hepatic gluconeogenesis increases, thereby causing elevated blood glucose and hence increased HbA_1c_ levels over time. Prolonged hyperglycemia can cause oxidative stress in β cells.^35^ E2 can prevent acute oxidative injury in β-cells in a hyperglycemic state by suppressing the β-cell translocation gene 2 (BTG_2_)-p53-Bax pathway.^36^ ERα localization in pancreatic β cells shows that E2 can confer protective effects against oxidative stress directly on β cells^37^ and additionally in hepatocytes^38^ to prevent insulin-deficient diabetes. A meta-analysis showed women undergoing HRT had alterations in metabolic syndrome components,^39^ thereby supporting that perturbations in sex hormone levels can impair glucose homeostasis. These observations, together with mechanistic evidence, are consistent and support our results.

### Strengths and limitations

To our knowledge, this study is the first population-based study to evaluate the relations between endogenous 17-OHP and glucose metabolism in both men and women. We have a relatively large sample size for the cross-sectional analyses from a well-characterized population-based study in men and women. This allowed us to adjust for numerous potential confounders. Another strength of this study is the prospective design with OGTT data available at both baseline and follow-up, allowing us to investigate not only the development of clinically diagnosed T2D but also of early derangements in glucose metabolism and newly OGTT-diagnosed T2D. However, this study also has limitations. While we adjusted our results for many established T2D risk factors, we did not have detailed dietary information, and the possibility of residual confounding cannot be precluded. Additionally, in the cross-sectional analyses, we cannot clearly distinguish cause and effect. Also, we could not identify women with polycystic ovarian syndrome (PCOS) in our dataset as the information is unavailable. PCOS symptoms persist even in postmenopausal women and could cause perturbations in sex hormone concentrations and, thus, metabolic processes. Lastly, we could not account for the effects of change in endogenous progestogens and estrogens, as the sex hormones were measured only at baseline.

## Conclusions

Our findings support an inter-relation between endogenous female sex hormones and altered glycemic metabolism not only in middle-aged and elderly women but also in men. However, future studies should corroborate our findings in both men and women, in well-powered settings, with sufficient follow-up, and investigate directional associations through Mendelian randomization.

10.1136/bmjdrc-2020-001951.supp3 Supplementary data
