# Supplementary material for: Cross-sectional and prospective relationships of endogenous progestogens and estrogens with glucose metabolism in men and women: a KORA F4/FF4 Study
Source: BMJ Open Diabetes Res Care. 2021 Feb 11;9(1):e001951. doi: 10.1136/bmjdrc-2020-001951 (PMC7880095; doi:10.1136/bmjdrc-2020-001951)
Supplement: Supplementary data [file bmjdrc-2020-001951supp001.pdf]

Supplementary Figure 1 – Ascertainment of the glycemic deterioration outcome in men and women of KORA F4/FF4.

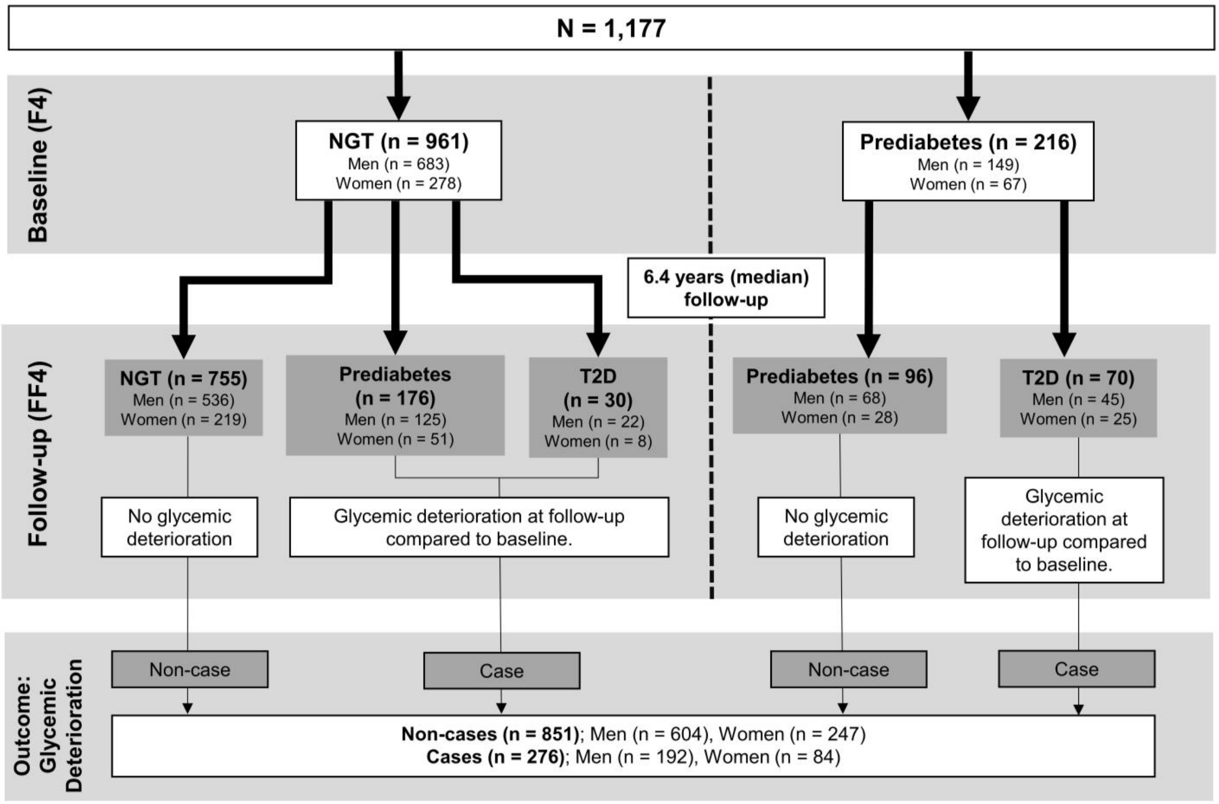

Illustration of the classification of participants with glycemic deterioration over the follow-up period. Non-cases were those who had normal glucose tolerance (NGT) at baseline (F4) and at follow-up (FF4) or who had prediabetes at baseline (F4) and follow-up (FF4). Cases were those who progressed from NGT at baseline to either prediabetes or T2D at follow-up or who progressed from prediabetes at baseline to T2D at follow-up.
