# Supplementary material for: Cross-sectional and prospective relationships of endogenous progestogens and estrogens with glucose metabolism in men and women: a KORA F4/FF4 Study
Source: BMJ Open Diabetes Res Care. 2021 Feb 11;9(1):e001951. doi: 10.1136/bmjdrc-2020-001951 (PMC7880095; doi:10.1136/bmjdrc-2020-001951)
Supplement: Supplementary data [file bmjdrc-2020-001951supp012.pdf]

Supplementary Materials and Methods 1 – Steroid Quantification of Hormones in Serum Samples

Steroid quantification in serum was performed at the Metabolomics Platform of the Genome Analysis Center, Helmholtz-Zentrum München. 19 steroids were quantified using an extended version of the Absolute*IDQ*™ Stero17 Kit and LC-ESI-MS/MS: aldosterone, androstenedione (androst-4-en-3,17-dione), androsterone, corticosterone, cortisol, cortisone, 11-deoxycorticosterone, 11-deoxycortisol, dehydroepiandrosterone (DHEA), dehydroepiandrosterone sulfate (DHEAS), dihydrotestosterone (DHT), estradiol (E2), estrone, etiocholanolone, 17α-hydroxyprogesterone, progesterone, testosterone, pregnenolone, pregnanediol (the last two steroids were assessed semi-quantitatively). Compound identification and quantification were based on scheduled multiple reaction monitoring measurements (sMRM). Sample preparation and LC-MS/MS measurements were performed as described by the manufacturer in manual UM-STERO17 [1]. Absolute*IDQ*™ Stero17 Kit methodology conforms to the "Guideline on Bioanalytical Method Validation" outlined by the EMEA [2], implying proof of reproducibility within a given error range. Analytical specification for LOD (limit of detection), LLOQ, and ULOQ (lower and upper limit of quantification), specificity, linearity, precision, accuracy, reproducibility, and stability were determined experimentally by Biocrates and are described in the manual AS-STERO17. A detailed method description has been published [3]. Method implementation in the laboratory is described as below:

Serum samples have been prepared in the group of the collaboration partner. All samples have been stored at -80°C until sample preparation for measurements. In the laboratory, 400 µl of ultrapure water were pipetted into each well of a 2 ml 96-well deep well plate. 20 µl of the internal standard mix was added to each well, except the blank cell. Thereafter, 250 µl of blank, calibration standards, quality control samples, and serum samples were pipetted into the distinct respective wells. The well contents were mixed by aspiration using robot-driven pipets. In between, the SPE (solid phase extraction) plate of the kit was conditioned successively with 1 ml of dichloromethane, followed by 1 ml acetonitrile, 1 ml methanol, and 1 ml ultrapure water. Except for sample loading, all SPE purification steps (conditioning, washing, drying, and eluting) were done by pressing solvents through the SPE plate using nitrogen and the positive pressure unit. The velocity was regulated by variation of the nitrogen pressure. After plate conditioning, the mixed samples were loaded onto the SPE plate. The samples dropped through very slowly by gravitation (1-2 drops per second). The SPE plate was washed with 500 µl water, dried for 1 h under nitrogen stream (58 psi). Steroids were subsequently eluted in two steps: 1) Two times with 500 µl

dichloromethane into the same deep well plate (all steroids except DHEAS eluted), the eluate was dried each time for 20 min at 45 psi. 2) With 600 µl acetonitrile into another deep well plate. The first dichloromethane fraction was dissolved in 50 µl of methanol/water (25/75 v/v) and the plate was covered with a lid. To facilitate dissolving, the plate was treated for 1 min in an ultrasonic bath and afterward shaken for 5 min at 600 rpm. The second acetonitrile fraction was diluted with 400 µl of water and after covering the plate was treated like the dichloromethane fraction. Both plates were centrifuged at 50 x *g* and placed into the cooled autosampler (10 °C) for LC-MS/MS measurements. The LC-separation of both fractions was performed using 470 ml ultrapure water and the content of three ampules of the kit as mobile phase A and acetonitrile/methanol/ultrapure water v/v/v 85/10/5 as mobile phase B. Steroids were separated on the HPLC column for Absolute*IDQ*™ Stero17 Kit combined with the precolumn SecurityGuard Cartridge C18 4 x 2 mm (for HPLC, Phenomenex Cat No. AJ0-4286).

HPLC grade solvents were used for sample preparation and measurements. Samples were handled using a Hamilton Microlab STAR™ robot (Hamilton Bonaduz AG, Bonaduz, Switzerland) and a Waters Positive Pressure-96 Processor (Waters GmbH, Eschborn, Germany), besides standard laboratory equipment. Mass spectrometric analyses were using a QTRAP 5500 triple quadrupole system (Sciex Deutschland GmbH, Darmstadt, Germany) equipped with a 1260 Series HPLC (Agilent Technologies Deutschland GmbH, Böblingen, Germany) and a HTC PAL autosampler (CTC Analytics, Zwingen, Switzerland) controlled by the software Analyst 1.6.2. Data evaluation for quantification of metabolite concentrations and quality assessment was performed with the software MultiQuant 3.0.1 (Sciex) and the Met*IDQ*™ software package, which is an integral part of the Absolute*IDQ*™ Stero17 Kit. Metabolite concentrations were calculated using internal standards and reported in nM or ng/ml.

References

[1] BIOCRATES Life Sciences. Absolute*IDQ*™ Stero17 Kit. Increased confidence in steroid hormone analysis. https://biocrates.com/wp-content/uploads/2020/02/Biocrates_Stero17.pdf; 2019. [accessed 6 October 2020]

[2] Committee for Medicinal Products for Human Use (CHMP). Guideline on bioanalytical method validation. EMEA/CHMP/EWP/192217/2009 Rev. 1 Corr. 2. https://www.ema.europa.eu/en/documents/scientific-guideline/guideline-bioanalytical-method-validation_en.pdf; 2011. [accessed 6 October 2020]

[3] Breier M, Wahl S, Prehn C, Ferrari U, Sacco V, Weise M, et al. Immediate reduction of serum citrulline but no change of steroid prodile after initiation of metformin in individuals with type 2 diabetes. J Steroid Biochem. 2017; 174;114-9. https://doi.org/10.1016/j.jsbmb.2017.08.004

[4] Rinaldi S, Geay A, Déchaud H, Biessy C, Zeleniuch-Jacquotte A, Akhmedkhanov A, et al. Validity of free testosterone and free estradiol determinations in serum samples from postmenopausal women by theoretical calculations. Cancer Epidemiol Biomarkers Prev. 2002;11:1065-71. https://cebp.aacrjournals.org/content/11/10/1065.long
