# Supplementary material for: Cross-sectional and prospective relationships of endogenous progestogens and estrogens with glucose metabolism in men and women: a KORA F4/FF4 Study
Source: BMJ Open Diabetes Res Care. 2021 Feb 11;9(1):e001951. doi: 10.1136/bmjdrc-2020-001951 (PMC7880095; doi:10.1136/bmjdrc-2020-001951)
Supplement: Supplementary data [file bmjdrc-2020-001951supp013.pdf]

Supplementary Methods and Materials 2 – Data pre-processing of 17-OHP, Progesterone, and Total E2 measurements

The methods below were used for NA imputation and removal, normalizing the measurements to their plate effects, as well as for calibrating for batch effects for measurements of 17-OHP, progesterone, and total E2, where necessary.

i) NA removal and imputation

NA detection was conducted and removal was performed if more than 40% of steroids of all samples were labeled "NA" or "0" in the raw data set. For the raw data set, only estrone was detected to fulfill these criteria and was therefore subsequently removed from the data set. All other steroids were below 40% NA. These remaining NA or zero values were imputed by a minimum values replacement algorithm, implemented as follows: The minimum value - which could be measured for each metabolite from all plates combined - is gathered. This value is not allowed to be equal to zero as the export from the MetIDQ-System, in which the raw measurement data is translated to quantitative data, unfortunately, uses "NA" and "0" synonymously. To mitigate the effects which could be incurred by using these minimum values directly as imputation values, further steps are to be undertaken.

1. The minimum values are not used directly: they are divided by √2 to emulate their real concentrations being well below the minimally detected ones;
2. These newly calculated values, called "replacers", are then permuted randomly in a range of 0.75*replacer up to 1.25*replacer to mitigate the statistical effects of repeating the same number over and over again.

ii) Plate normalization

For each plate and metabolite, plate specific mean values ("plate means") are calculated. For this, the concentrations of each metabolite of the QC-2 samples (n = 5) are used. The plate means are then used to calculate an overall mean of all plates. These steps have to be performed for all metabolites (x_1, x_2, x_3, …, x_n) on all plates (n_1, n_2, n_3, …, n_j) of the data set.

$$Platemean[metabolite(x)] = \frac{\sum C[metabolite(x)]}{N}$$

Where N = number of reference samples and C = concentration.

These plate means are then used to calculate the overall "means of all plates". This has to be calculated again for all metabolites (x_1, x_2, x_3, …, x_n) for each plate in the data set.

$$Overallmean[X] = \frac{\sum Means[X]}{N}$$

Where X = metabolite (x) of plates ($n \ldots n_j$) and N = number of plates ($n \ldots n_j$)

The plate factors are calculated for each plate separately, e.g. for plate 1 as follows:

$$Factor[Y] = \frac{Overallmean[X]}{Mean[Y]}$$

Where X = metabolite (X) of Plates and Y = Plate(n)[metabolite (X)]

In the final step of normalization, each metabolite concentration is multiplied with their corresponding plate factors, e.g. metabolite concentration of each sample on plate 1 is multiplied with plate factor 1.

$$Normalised[Y] = Factor[Y] \times Concentration[Y]$$

Where Y = Plate(n)[metabolite (X)]

In the last step, the duplicates from Batch 1 of the measurements are removed by averaging them.

ii) Calibration of batch effects of 17-OHP, Progesterone, and E2

Regarding measurements of 17-OHP, progesterone, and E2, serum samples from all KORA F4 participants were initially measured between January and November 2013 (batch 1). Due to measurement problems, they had to be repeated for 980 serum samples with the same methods described above between July 2017 and March 2018 (batch 2). To avoid batch effects, we used 175 duplicate measurements from the same participants of batches 1 and 2 to develop calibration formulas. The intercept and slope of Passing-Bablok regressions were used to calibrate batch 2 measurements with measures from batch 1.

References

[1] BIOCRATES Life Sciences. Absolute*IDQ*™ Stero17 Kit. Increased confidence in steroid hormone analysis. https://biocrates.com/wp-content/uploads/2020/02/Biocrates_Stero17.pdf; 2019. [accessed 6 October 2020]

[2] Committee for Medicinal Products for Human Use (CHMP). Guideline on bioanalytical method validation. EMEA/CHMP/EWP/192217/2009 Rev. 1 Corr. 2. https://www.ema.europa.eu/en/documents/scientific-guideline/guideline-bioanalytical-method-validation_en.pdf; 2011. [accessed 6 October 2020]

[3] Breier M, Wahl S, Prehn C, Ferrari U, Sacco V, Weise M, et al. Immediate reduction of serum citrulline but no change of steroid prodile after initiation of metformin in individuals with type 2 diabetes. J Steroid Biochem. 2017; 174;114-9. https://doi.org/10.1016/j.jsbmb.2017.08.004

[4] Rinaldi S, Geay A, Déchaud H, Biessy C, Zeleniuch-Jacquotte A, Akhmedkhanov A, et al. Validity of free testosterone and free estradiol determinations in serum samples from postmenopausal women by theoretical calculations. Cancer Epidemiol Biomarkers Prev. 2002;11:1065-71. https://cebp.aacrjournals.org/content/11/10/1065.long
