# Supplementary material for: Cross-sectional and prospective relationships of endogenous progestogens and estrogens with glucose metabolism in men and women: a KORA F4/FF4 Study
Source: BMJ Open Diabetes Res Care. 2021 Feb 11;9(1):e001951. doi: 10.1136/bmjdrc-2020-001951 (PMC7880095; doi:10.1136/bmjdrc-2020-001951)
Supplement: Supplementary data [file bmjdrc-2020-001951supp014.pdf]

Supplementary Methods and Materials 3 – Calculation of Free E2 (fE2)

Free E2 was calculated using mass action equations. The formula is as follows: [fE2] = [E2] – N – K_aSHBG + √(n + [SHBG] – $[E2]^2$ + (4 × N × [E2])) / 2×N × K_aSHBG, where [fE2] is the concentration of free E2, [E2] is the concentration of E2, N = (0.5217 × ([albumin] × K_aAlb) + 1), [albumin] is the concentration of albumin, K_aAlb is the association constant of E2 to albumin (4.2 x 10^4 nmol/L), and K_aSHBG is the association constant of E2 to SHBG (3.4× 10^8 nmol/L) [4].

References

[1] BIOCRATES Life Sciences. Absolute*IDQ*™ Stero17 Kit. Increased confidence in steroid hormone analysis. https://biocrates.com/wp-content/uploads/2020/02/Biocrates_Stero17.pdf; 2019. [accessed 6 October 2020]

[2] Committee for Medicinal Products for Human Use (CHMP). Guideline on bioanalytical method validation. EMEA/CHMP/EWP/192217/2009 Rev. 1 Corr. 2. https://www.ema.europa.eu/en/documents/scientific-guideline/guideline-bioanalytical-method-validation_en.pdf; 2011. [accessed 6 October 2020]

[3] Breier M, Wahl S, Prehn C, Ferrari U, Sacco V, Weise M, et al. Immediate reduction of serum citrulline but no change of steroid prodile after initiation of metformin in individuals with type 2 diabetes. J Steroid Biochem. 2017; 174;114-9. https://doi.org/10.1016/j.jsbmb.2017.08.004

[4] Rinaldi S, Geay A, Déchaud H, Biessy C, Zeleniuch-Jacquotte A, Akhmedkhanov A, et al. Validity of free testosterone and free estradiol determinations in serum samples from postmenopausal women by theoretical calculations. Cancer Epidemiol Biomarkers Prev. 2002;11:1065-71. https://cebp.aacrjournals.org/content/11/10/1065.long
