# Supplementary material for: Cross-sectional and prospective relationships of endogenous progestogens and estrogens with glucose metabolism in men and women: a KORA F4/FF4 Study
Source: BMJ Open Diabetes Res Care. 2021 Feb 11;9(1):e001951. doi: 10.1136/bmjdrc-2020-001951 (PMC7880095; doi:10.1136/bmjdrc-2020-001951)
Supplement: Supplementary data [file bmjdrc-2020-001951supp004.pdf]

Supplementary Table 1 – P-values for sex interactions in associations between endogenous progestogens and estrogens with glycemic traits and glycemic deterioration in the KORA F4/FF4 study.

	P-value for sex interaction			
	**17-OHP**	**Progesterone**	**E2**	**Free E2**
**Fasting glucose**	0.095	0.445	0.306	0.139
**2h-glucose**	0.829	0.860	0.799	0.506
**HbA_{1c}**	0.545	0.929	0.714	0.051
**Fasting insulin**	**0.004**	0.149	**0.027**	**0.008**
**QUICKI**	**0.005**	0.104	**0.036**	**0.007**
**Glycemic deterioration**	0.152	0.412	0.621	0.547

P-values for interaction between sex and sex hormones on continuous glycemic outcomes in cross-sectional linear regression models and glycemic deterioration in prospective logistic regression models. Adjusted for baseline age, waist circumference, height, triglycerides, total cholesterol/HDL-cholesterol ratio), hypertension, statin use (model 1), smoking, alcohol consumption, physical activity, CRP, eGFR, TSH, and parental diabetes history (model 2). Sex interaction was entered as a multiplicative term in model 2.
