# Supplementary material for: Cross-sectional and prospective relationships of endogenous progestogens and estrogens with glucose metabolism in men and women: a KORA F4/FF4 Study
Source: BMJ Open Diabetes Res Care. 2021 Feb 11;9(1):e001951. doi: 10.1136/bmjdrc-2020-001951 (PMC7880095; doi:10.1136/bmjdrc-2020-001951)
Supplement: Supplementary data [file bmjdrc-2020-001951supp005.pdf]

Supplementary Table 2 - Cross-sectional associations of endogenous progestogens and estrogens with glycemic traits in men of KORA F4.

		17-OHP	Progesterone	Progesterone[a]	E2	E2[b]	fE2
		β (95% CI)	β (95% CI)	β (95% CI)	β (95% CI)	β (95% CI)	β (95% CI)
Fasting Glucose	Model 1	-0.019 (-0.064 – 0.026) P = 0.410	0.017 (-0.025 – 0.059) P = 0.438	-	-0.014 (-0.063 – 0.035) P = 0.570	-	0.038 (-0.014 – 0.090) P = 0.153
	Model 2	-0.014 (-0.058 – 0.030) P = 0.529	0.013 (-0.028 – 0.054) P = 0.544	0.011 (-0.030 – 0.051) P = 0.606	0.002 (-0.046 – 0.049) P = 0.947	0.003 (-0.047 – 0.052) P = 0.913	0.003 (-0.048 – 0.054) P = 0.920
2h-glucose	Model 1	**-0.074 (-0.130 – -0.019) P = 0.009**	-0.024 (-0.075 – 0.027) P= 0.354	-	**-0.059 (-0.118 – -0.001) P = 0.048**	-	0.039 (-0.024 – 0.103) P = 0.225
	Model 2	**-0.067 (-0.120 – -0.013) P = 0.014**	-0.029 (-0.078 – 0.020) P = 0.242	-0.030 (-0.079 – 0.019) P = 0.228	-0.024 (-0.081 – 0.033) P = 0.413	-0.013 (-0.073 – 0.046) P = 0.660	0.016 (-0.046 – 0.078) P = 0.609
HbA_{1c}	Model 1	-0.041 (-0.087 – 0.006) P = 0.088	0.021 (-0.022 – 0.065) P = 0.340	-	-0.048 (-0.098 – 0.002) P = 0.061	-	**0.086 (0.032 – 0.140) P = 0.002**
	Model 2	-0.032 (-0.078 – 0.013) P = 0.163	0.025 (-0.017 – 0.067) P = 0.248	0.028 (-0.015 – 0.070) P = 0.199	-0.024 (-0.073 – 0.026) P = 0.350	0.012 (-0.039 – 0.063) P = 0.648	**0.079 (0.027 – 0.132) P = 0.003**
Fasting Insulin	Model 1	**-0.093 (-0.140 – -0.046) P < 0.001**	**-0.052 (-0.096 – -0.008) P = 0.020**	-	**-0.113 (-0.163 – -0.062) P < 0.001**	-	0.013 (-0.041 – 0.068) P = 0.628
	Model 2	**-0.074 (-0.118 – -0.030) P = 0.001**	**-0.045 (-0.086 – -0.004) P = 0.031**	**-0.047 (-0.088 – -0.006) P = 0.026**	**-0.068 (-0.116 – -0.020) P = 0.006**	**-0.055 (-0.105 – -0.005) P = 0.030**	-0.008 (-0.059 – 0.044) P= 0.771
QUICKI	Model 1	**0.079 (0.032 – 0.126) P = 0.001**	**0.045 (0.001 – 0.088) P = 0.046**	-	**0.105 (0.054 – 0.155) P < 0.001**	-	-0.031 (-0.085 – 0.024) P = 0.271
	Model 2	**0.061 (0.018 – 0.105) P = 0.006**	0.040 (-0.001 – 0.080) P = 0.057	**0.041 (0.001 – 0.082) P = 0.046**	**0.059 (0.012 – 0.107) P = 0.015**	0.044 (-0.005 – 0.093) P = 0.079	-0.004 (-0.055 – 0.047) P = 0.888

All results are from multivariate linear regression models. Adjusted for baseline age, waist circumference, height, triglycerides, total cholesterol/HDL-cholesterol ratio), hypertension, statin use (model 1), smoking, alcohol consumption, physical activity, CRP, eGFR, TSH, and parental diabetes history (model 2). Effect estimates with 95% CIs were calculated for a one sex-specific SD increase on the log scale of progestogen and estrogen levels, respectively. Significant results are printed in bold. Abbreviations: 17-OHP: 17α-hydroxyprogesterone, CRP: C-reactive protein, eGFR: Estimated glomerular filtration rate, SHBG: Sex hormone-binding globulin, TSH: Thyroid-stimulating hormone.

[a] *Models were additionally adjusted for albumin*

[b] *Models were additionally adjusted for SHBG.*

Lau LHY, *et al. BMJ Open Diab Res Care* 2021; 9:e001951. doi: 10.1136/bmjdrc-2020-001951
