# Supplementary material for: Cross-sectional and prospective relationships of endogenous progestogens and estrogens with glucose metabolism in men and women: a KORA F4/FF4 Study
Source: BMJ Open Diabetes Res Care. 2021 Feb 11;9(1):e001951. doi: 10.1136/bmjdrc-2020-001951 (PMC7880095; doi:10.1136/bmjdrc-2020-001951)
Supplement: Supplementary data [file bmjdrc-2020-001951supp006.pdf]

Supplementary Table 3 - Cross-sectional associations of endogenous progestogens and estrogens with glycemic traits in women of KORA F4.

		17-OHP	Progesterone	Progesterone[a]	E2	E2[b]	fE2
		β (95% CI)	β (95% CI)	β (95% CI)	β (95% CI)	β (95% CI)	β (95% CI)
**Fasting Glucose**	**Model 1**	**0.071 (0.015 – 0.127) P = 0.013**	0.042 (-0.015 – 0.099) P = 0.148	-	0.049 (-0.008 – 0.106) P = 0.093	-	**0.094 (0.033 – 0.156) P = 0.003**
	**Model 2**	**0.068 (0.014 – 0.123) P = 0.014**	0.049 (-0.006 – 0.105) P = 0.081	0.054 (-0.001 – 0.109) P = 0.054	0.052 (-0.004 – 0.108) P = 0.067	0.055 (-0.000 – 0.111) P = 0.051	**0.080 (0.020 – 0.141) P = 0.009**
	**Model 2 (Sens.)[c]**	**0.098 (0.032 – 0.164) P = 0.004**	**0.071 (0.007 – 0.136) P = 0.031**	-	**0.076 (0.014 – 0.137) P = 0.016**	-	**0.092 (0.026 – 0.159) P = 0.007**
**2h-glucose**	**Model 1**	-0.056 (-0.130 – 0.017) P = 0.131	-0.050 (-0.124 – 0.025) P = 0.190	-	-0.001 (-0.076 – 0.074) P = 0.974	-	0.028 (-0.053 – 0.108) P = 0.502
	**Model 2**	-0.053 (-0.125 – 0.019) P = 0.148	-0.040 (-0.114 – 0.033) P = 0.278	-0.036 (-0.109 – 0.037) P = 0.334	0.001 (-0.073 – 0.074) P = 0.987	0.001 (-0.072 – 0.075) P = 0.975	0.014 (-0.065 – 0.094) P = 0.725
	**Model 2 (Sens.)[c]**	-0.057 (-0.145 – 0.031) P= 0.201	-0.030 (-0.117 – 0.056) P = 0.492	-	0.017 (-0.065 – 0.098) P = 0.690	-	0.018 (-0.070 – 0.107) P = 0.682
**HbA_{1c}**	**Model 1**	-0.022 (-0.076 – 0.033) P = 0.438	0.032 (-0.023 – 0.087) P = 0.252	-	0.004 (-0.052 – 0.060) P = 0.888	-	**0.123 (0.064 – 0.182) P < 0.001**
	**Model 2**	-0.020 (-0.074 – 0.035) P = 0.481	0.040 (-0.015 – 0.095) P = 0.154	0.040 (-0.015 – 0.095) P = 0.150	0.008 (-0.048 – 0.064) P = 0.777	0.019 (-0.034 – 0.072) P = 0.481	**0.121 (0.062 – 0.180) P < 0.001**
	**Model 2 (Sens.)[c]**	0.007 (-0.057 – 0.071) P = 0.832	**0.071 (0.008 – 0.133) P = 0.026**	-	0.033 (-0.026 – 0.093) P = 0.271	-	**0.139 (0.076 – 0.202) P < 0.001**
**Fasting Insulin**	**Model 1**	-0.002 (-0.059 – 0.056) P = 0.959	-0.033 (-0.092 – 0.026) P = 0.268	-	-0.035 (-0.094 – 0.024) P = 0.244	-	0.008 (-0.056 – 0.071) P = 0.810
	**Model 2**	-0.003 (-0.059 – 0.052) P = 0.907	-0.031 (-0.087 – 0.025) P = 0.279	-0.027 (-0.083 – 0.029) P = 0.343	-0.033 (-0.090 – 0.024) P = 0.260	-0.030 (-0.088 – 0.027) P = 0.298	-0.003 (-0.065 – 0.058) P = 0.913
	**Model 2 (Sens.)[c]**	-0.005 (-0.072 – 0.062) P = 0.884	-0.032 (-0.097 – 0.033) P = 0.336	-	-0.013 (-0.075 – 0.050) P = 0.692	-	0.008 (-0.059 – 0.075) P = 0.814
**QUICKI**	**Model 1**	-0.021 (-0.079 – 0.036) P = 0.466	0.015 (-0.045 – 0.075) P = 0.625	-	0.018 (-0.043 – 0.078) P = 0.568	-	-0.033 (-0.098 – 0.032) P = 0.316
	**Model 2**	-0.020 (-0.074 – 0.035) P = 0.486	0.010 (-0.047 – 0.066) P = 0.742	0.004 (-0.052 – 0.061) P = 0.879	0.014 (-0.043 – 0.072) P = 0.622	0.011 (-0.047 – 0.068) P = 0.713	-0.018 (-0.080 – 0.044) P = 0.562
	**Model 2 (Sens.)[c]**	-0.027 (-0.094 – 0.040) P = 0.433	0.005 (-0.060 – 0.071) P = 0.877	-	-0.012 (-0.074 – 0.051) P = 0.717	-	-0.034 (-0.101 – 0.034) P = 0.327

All results are from multivariate linear regression models. Adjusted for baseline age, waist circumference, height, triglycerides, total cholesterol/HDL-cholesterol ratio, hypertension, statin use (model 1), smoking, alcohol consumption, physical activity, CRP, eGFR, TSH, and parental diabetes history (model 2). Effect estimates with 95% CIs were calculated for a one sex-specific SD increase on the log scale of progestogen and estrogen levels, respectively. Significant results are printed in bold. Abbreviations: 17-OHP: 17α-hydroxyprogesterone, CRP: C-reactive protein, eGFR: Estimated glomerular filtration rate, SHBG: Sex hormone-binding globulin, TSH: Thyroid-stimulating hormone.

[a] *Models were additionally adjusted for albumin*
[b] *Models were additionally adjusted for SHBG.*
[c] *Sensitivity analyses: Perimenopausal women excluded (n = outcome specific).*

Lau LHY, *et al. BMJ Open Diab Res Care* 2021; 9:e001951. doi: 10.1136/bmjdrc-2020-001951
