# Supplementary material for: Cross-sectional and prospective relationships of endogenous progestogens and estrogens with glucose metabolism in men and women: a KORA F4/FF4 Study
Source: BMJ Open Diabetes Res Care. 2021 Feb 11;9(1):e001951. doi: 10.1136/bmjdrc-2020-001951 (PMC7880095; doi:10.1136/bmjdrc-2020-001951)
Supplement: Supplementary data [file bmjdrc-2020-001951supp009.pdf]

Supplementary Table 6 – P-values for interaction terms between 17-OHP and progesterone in multivariate linear regression models examining associations between sex hormones and glycemic traits in men and women of the KORA F4/FF4 cohort

T2D-related Traits	P for interaction between 17-OHP & progesterone		
	Men	Women	Women**
**Fasting Glucose**	-	0.882	0.351
**2h-glucose**	0.510	-	-
**HbA_{1c}**	-	-	0.733***
**Fasting Insulin**	**0.002**	-	-
**QUICKI**	**0.008**	-	-

All results are from a multivariate linear regression. Interactions between 17-OHP and progesterone were examined by adding a multiplicative term into model 2. Interactions were calculated only for significant associations between exposure and respective outcomes. *Adjusted for baseline age, waist circumference, height, triglycerides, total cholesterol/HDL-cholesterol ratio, hypertension, statin use, smoking, alcohol consumption, physical activity, CRP, eGFR (creatinine-based), TSH, and parental diabetes history. **Sensitivity analyses performed where perimenopausal women (n = outcome specific) were excluded. ***Interaction analysis performed due to significant associations between progesterone and HbA_{1c} upon exclusion of perimenopausal women (n = 66). Significant results are printed in bold. Abbreviations: 17-OHP: 17α-hydroxyprogesterone, CRP: C-reactive protein, eGFR: Estimated glomerular filtration rate, SHBG: Sex hormone-binding globulin, TSH: Thyroid-stimulating hormone.*
