# Supplementary material for: Cross-sectional and prospective relationships of endogenous progestogens and estrogens with glucose metabolism in men and women: a KORA F4/FF4 Study
Source: BMJ Open Diabetes Res Care. 2021 Feb 11;9(1):e001951. doi: 10.1136/bmjdrc-2020-001951 (PMC7880095; doi:10.1136/bmjdrc-2020-001951)
Supplement: Supplementary data [file bmjdrc-2020-001951supp007.pdf]

Supplementary Table 4 – Prospective associations of endogenous progestogens and estrogens with glycemic deterioration.

		Men		Women	
		**Glycemic Deterioration** *Cases (n = 193), non-cases (n = 604)*		**Glycemic Deterioration** *Cases (n = 85), non-cases (n = 247)*	
		**OR (95% CI)**	**P value**	**OR (95% CI)**	**P-value**
**17-OHP**	Model 1	0.969 (0.802 – 1.172)	0.746	1.255 (0.950 – 1.661)	0.110
	Model 2	1.005 (0.824 – 1.226)	0.961	1.358 (0.999, 1.853)	0.051
	Model 2 (Sens.)*	-	-	**1.518 (1.033, 2.264)**	**0.036**
**Progesterone**	Model 1	0.934 (0.795 – 1.105)	0.413	0.983 (0.756 – 1.296)	0.902
	Model 2	0.929 (0.782 – 1.112)	0.412	1.066 (0.795 – 1.445)	0.675
	Model 2 + Albumin**	0.926 (0.779 – 1.108)	0.388	1.043 (0.778 – 1.414)	0.780
	Model 2 (Sens.)*	-	-	1.168 (0.813 – 1.725)	0.414
**E2**	Model 1	0.899 (0.750 – 1.078)	0.246	0.865 (0.671 – 1.128)	0.267
	Model 2	0.910 (0.751 – 1.104)	0.337	0.879 (0.664 – 1.175)	0.371
	Model 2 + SHBG**	0.909 (0.743 – 1.112)	0.352	0.890 (0.668 – 1.199)	0.431
	Model 2 (Sens.)*	-	-	0.893 (0.662 – 1.219)	0.465
**fE2**	Model 1	0.970 (0.797 – 1.183)	0.760	0.907 (0.664 – 1.245)	0.542
	Model 2	0.922 (0.747 – 1.138)	0.448	0.901 (0.647 – 1.258)	0.535
	Model 2 (Sens.)*	-	-	0.851 (0.592 – 1.223)	0.380

*All results are from a multivariate logistic regression. Model 1: Adjusted for baseline age, waist circumference, and height. Model 2: Model 1 + triglycerides, total cholesterol/HDL-cholesterol ratio, hypertension, statin use, smoking, alcohol consumption, physical activity, CRP, eGFR (creatinine-based), TSH, and parental diabetes history. ORs with 95% CIs for worse glycemic outcomes were calculated for a one sex-specific SD increase on the log scale of progestogen and estrogen levels, respectively.*Sensitivity analysis where perimenopausal women (n = 66; 15 cases and 51 non-cases) were excluded. **Further adjustment of progesterone with albumin and E2 with SHBG. Abbreviations: 17-OHP: 17α-hydroxyprogesterone, CRP: C-reactive protein, eGFR: Estimated glomerular filtration rate, TSH: Thyroid-stimulating hormone.*

Lau LHY, *et al. BMJ Open Diab Res Care* 2021; 9:e001951. doi: 10.1136/bmjdrc-2020-001951
