# Supplementary material for: Cross-sectional and prospective relationships of endogenous progestogens and estrogens with glucose metabolism in men and women: a KORA F4/FF4 Study
Source: BMJ Open Diabetes Res Care. 2021 Feb 11;9(1):e001951. doi: 10.1136/bmjdrc-2020-001951 (PMC7880095; doi:10.1136/bmjdrc-2020-001951)
Supplement: Supplementary data [file bmjdrc-2020-001951supp002.pdf]

Supplementary Figure 2 – Non-linear associations of endogenous progestogens and estrogens with glycemic deterioration in men and women of KORA F4/FF4.

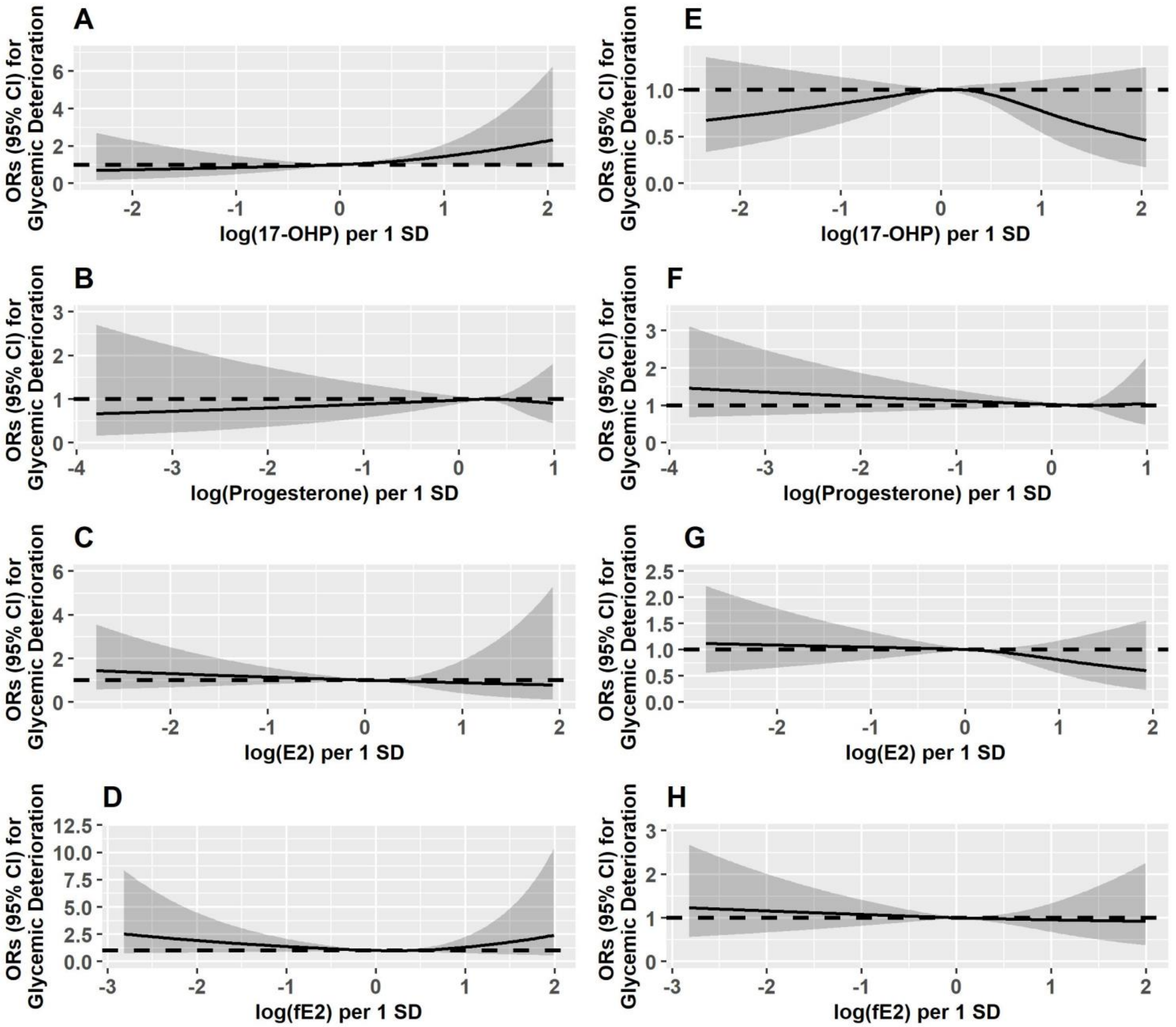

Non-linear relationships between sex hormones and glycemic deterioration in men (A, B, C, and D) and women (E, F, G, and H). Multivariate adjusted logistic regression models exploring sex hormone levels and associations with glycemic deterioration were entered into the model as a restricted cubic spline. Knots were placed at the 30th, 60th, and 90th percentiles. The shaded grey area represents the 95% CI of the spline estimation. *Adjusted for baseline age, waist circumference, height, triglycerides, total cholesterol/HDL-cholesterol ratio, hypertension, statin use, smoking, alcohol consumption, physical activity, CRP, eGFR (creatinine-based), TSH, and parental diabetes history. Additionally, models for progesterone are adjusted for albumin, and models for E2 are adjusted for SHBG. Abbreviations: 17-OHP: 17α-hydroxyprogesterone, CRP: C-reactive protein, eGFR: Estimated glomerular filtration rate, SHBG: Sex hormone-binding globulin, TSH: Thyroid-stimulating hormone. P-values for spline fit are described in Supplementary Table 5.*

Lau LHY, *et al. BMJ Open Diab Res Care* 2021; 9:e001951. doi: 10.1136/bmjdrc-2020-001951
