# Supplementary material for: Cross-sectional and prospective relationships of endogenous progestogens and estrogens with glucose metabolism in men and women: a KORA F4/FF4 Study
Source: BMJ Open Diabetes Res Care. 2021 Feb 11;9(1):e001951. doi: 10.1136/bmjdrc-2020-001951 (PMC7880095; doi:10.1136/bmjdrc-2020-001951)
Supplement: Supplementary data [file bmjdrc-2020-001951supp008.pdf]

Supplementary Table 5 – Non-linear associations of endogenous progestogens and estrogens with glycemic deterioration.

Sex Hormones	P-value for non-linear fit		
	Men	Women	Women**
17-OHP	0.103	0.568	0.356
Progesterone	0.794	0.665	0.344
Total E2	0.464	0.997	0.696
Free E2	0.922	0.108	0.324

Multivariate adjusted logistic regression models exploring sex hormone levels and associations with glycemic deterioration were entered into the model as a restricted cubic spline. Knots were placed at the 30th, 60th, and 90th percentiles. The shaded grey area represents the 95% CI of the spline estimation. *Adjusted for age, waist circumference, height, triglycerides, total cholesterol/HDL-cholesterol ratio, hypertension, statin use, smoking, alcohol consumption, physical activity, CRP, eGFR (creatinine-based), TSH, and parental diabetes history. Additionally, models for progesterone are adjusted for albumin, and models for E2 are adjusted for SHBG. **Sensitivity analyses performed where perimenopausal women (n = 66; 15 cases and 51 non-cases) were excluded. Abbreviations: 17-OHP: 17α-hydroxyprogesterone, CRP: C-reactive protein, eGFR: Estimated glomerular filtration rate, SHBG: Sex hormone-binding globulin, TSH: Thyroid-stimulating hormone.*

Lau LHY, *et al. BMJ Open Diab Res Care* 2021; 9:e001951. doi: 10.1136/bmjdrc-2020-001951
