# Supplementary material for: Cross-sectional and prospective relationships of endogenous progestogens and estrogens with glucose metabolism in men and women: a KORA F4/FF4 Study
Source: BMJ Open Diabetes Res Care. 2021 Feb 11;9(1):e001951. doi: 10.1136/bmjdrc-2020-001951 (PMC7880095; doi:10.1136/bmjdrc-2020-001951)
Supplement: Supplementary data [file bmjdrc-2020-001951supp010.pdf]

Supplementary Table 7 - Prospective associations of endogenous progestogens and estrogens with markers of glycemic traits in men of KORA F4/FF4.

		17-OHP	Progesterone	Progesterone[a]	E2	E2[b]	fE2
		β (95% CI)	β (95% CI)	β (95% CI)	β (95% CI)	β (95% CI)	β (95% CI)
Fasting Glucose	Model 1	-0.017<br>(-0.055 – 0.022)<br>P = 0.393	0.021<br>(-0.015 – 0.056)<br>p = 0.260	-	-0.026<br>(-0.063 – 0.011)<br>P = 0.174	-	0.016<br>(-0.025 – 0.057)<br>P = 0.440
	Model 2	-0.019<br>(-0.057 – 0.020)<br>P = 0.342	0.019<br>(-0.017 – 0.055)<br>P = 0.302	0.018<br>(-0.018 – 0.054)<br>P = 0.320	-0.021<br>(-0.059 – 0.017)<br>P = 0.274	-0.010<br>(-0.050 – 0.030)<br>P = 0.621	0.014<br>(-0.028 – 0.056)<br>P = 0.511
2h-glucose	Model 1	0.009<br>(-0.052 – 0.070)<br>P = 0.765	-0.021<br>(-0.072 – 0.031)<br>P = 0.428	-	-0.011<br>(-0.070 – 0.047)<br>P = 0.701	-	0.032<br>(-0.033 – 0.097)<br>P = 0.339
	Model 2	0.013<br>(-0.048 – 0.074)<br>P = 0.682	-0.021<br>(-0.073 – 0.031)<br>P = 0.423	-0.021<br>(-0.073 – 0.031)<br>P = 0.426	-0.005<br>(-0.064 – 0.055)<br>p = 0.878	0.006<br>(-0.057 – 0.070)<br>P = 0.841	0.024<br>(-0.042 – 0.090)<br>P = 0.470
HbA_{1c}	Model 1	0.013<br>(-0.026 – 0.052)<br>P = 0.516	0.007<br>(-0.029 – 0.043)<br>P = 0.715	-	-0.012<br>(-0.050 – 0.026)<br>P = 0.542	-	-0.030<br>(-0.072 – 0.011)<br>P = 0.155
	Model 2	0.007<br>(-0.032 – 0.045)<br>P = 0.738	0.007<br>(-0.029 – 0.043)<br>P = 0.703	0.005<br>(-0.030 – 0.041)<br>P = 0.775	-0.000<br>(-0.038 – 0.038)<br>P = 0.993	-0.017<br>(-0.057 – 0.022)<br>P = 0.394	-0.028<br>(-0.069 – 0.014)<br>P = 0.190
Fasting Insulin	Model 1	-0.007<br>(-0.057 – 0.043)<br>P = 0.785	**0.052<br>(0.005 – 0.098)<br>P = 0.030**	-	-0.005<br>(-0.053 – 0.044)<br>P = 0.853	-	0.027<br>(-0.027 – 0.080)<br>P = 0.327
	Model 2	-0.013<br>(-0.062 – 0.037)<br>P = 0.617	0.044<br>(-0.002 – 0.091)<br>P = 0.062	0.043<br>(-0.003 – 0.090)<br>P = 0.067	0.005<br>(-0.044 – 0.054)<br>P = 0.835	0.012<br>(-0.039 – 0.063)<br>P = 0.652	0.020<br>(-0.033 – 0.074)<br>P = 0.455
QUICKI	Model 1	0.009<br>(-0.042 – 0.059)<br>P = 0.741	**-0.048<br>(-0.095 – -0.000)<br>P = 0.049**	-	0.006<br>(-0.044 – 0.055)<br>P = 0.827	-	-0.032<br>(-0.086 – 0.023)<br>P = 0.256
	Model 2	0.014<br>(-0.036 – 0.065)<br>P = 0.578	-0.040<br>(-0.088 – 0.007)<br>P = 0.095	-0.039<br>(-0.087 – 0.008)<br>P = 0.104	-0.006<br>(-0.056 – 0.044)<br>P = 0.820	-0.014<br>(-0.067 – 0.038)<br>P = 0.595	-0.026<br>(-0.081 – 0.029)<br>P = 0.354

All results are from multivariate linear regression models. Adjusted for baseline values of respective glycemic traits, age, waist circumference, height, triglycerides, total cholesterol/HDL ratio), hypertension, statin use (model 1), smoking, alcohol consumption, physical activity, CRP, eGFR, TSH, and parental diabetes history (model 2). Effect estimates with 95% CIs were calculated for a one sex-specific SD increase on the log scale of progestogen and estrogen levels, respectively. Significant results are printed in bold. Abbreviations: 17-OHP: 17α-hydroxyprogesterone, CRP: C-reactive protein, eGFR: Estimated glomerular filtration rate, SHBG: Sex hormone-binding globulin, TSH: Thyroid-stimulating hormone.

[a] Models were additionally adjusted for albumin.

[b] *Models were additionally adjusted for SHBG.*
