# Supplementary material for: Cross-sectional and prospective relationships of endogenous progestogens and estrogens with glucose metabolism in men and women: a KORA F4/FF4 Study
Source: BMJ Open Diabetes Res Care. 2021 Feb 11;9(1):e001951. doi: 10.1136/bmjdrc-2020-001951 (PMC7880095; doi:10.1136/bmjdrc-2020-001951)
Supplement: Supplementary data [file bmjdrc-2020-001951supp011.pdf]

Supplementary Table 8 - Prospective associations of endogenous progestogens and estrogens with glycemic traits in women of KORA F4/FF4.

		17-OHP	Progesterone	Progesterone[a]	E2	E2[b]	fE2
		β (95% CI)	β (95% CI)	β (95% CI)	β (95% CI)	β (95% CI)	β (95% CI)
Fasting Glucose	Model 1	0.012 (-0.050 – 0.073) P = 0.713	-0.010 (-0.072 – 0.051) P = 0.742	-	-0.008 (-0.071 – 0.055) P = 0.800	-	0.043 (-0.027 – 0.113) P = 0.230
	Model 2	0.005 (-0.057 – 0.066) P = 0.881	-0.011 (-0.074 – 0.052) P = 0.733	-0.015 (-0.078 – 0.047) P = 0.627	0.004 (-0.059 – 0.067) P = 0.900	0.005 (-0.058 – 0.068) P = 0.872	0.054 (-0.016 – 0.123) P = 0.133
	Model 2 (Sens.)[c]	0.007 (-0.070 – 0.085) P = 0.851	-0.008 (-0.086 – 0.070) P = 0.849	-	0.010 (-0.060 – 0.080) P = 0.784	-	0.054 (-0.016 – 0.123) P = 0.133
2h-glucose	Model 1	0.035 (-0.050 – 0.120) P = 0.421	-0.007 (-0.093 – 0.079) p = 0.878	-	-0.060 (-0.146 – 0.025) P = 0.165	-	-0.047 (-0.144 – 0.049) P = 0.336
	Model 2	0.036 (-0.049 – 0.121) P = 0.402	0.004 (-0.083 – 0.091) P = 0.923	0.004 (-0.084 – 0.091) P = 0.935	-0.057 (-0.143 – 0.029) P = 0.192	-0.054 (-0.140 – 0.032) P = 0.219	-0.039 (-0.136 – 0.058) P = 0.433
	Model 2 (Sens.)[c]	0.074 (-0.026 – 0.173) P = 0.147	0.043 (-0.056 – 0.142) P = 0.394	-	-0.029 (-0.118 – 0.061) P = 0.528	-	-0.013 (-0.114 – 0.088) P = 0.800
HbA_{1c}	Model 1	0.001 (-0.057 – 0.059) P = 0.970	-0.044 (-0.102 – 0.015) p = 0.141	-	0.019 (-0.041 – 0.078) P = 0.538	-	-0.010 (-0.076 – 0.056) P = 0.761
	Model 2	0.012 (-0.046 – 0.070) P = 0.679	-0.027 (-0.086 – 0.032) P = 0.373	-0.026 (-0.085 – 0.033) P = 0.391	0.024 (-0.035 – 0.083) P = 0.418	0.024 (-0.035 – 0.083) P = 0.423	-0.006 (-0.072 – 0.060) P = 0.853
	Model 2 (Sens.)[c]	-0.004 (-0.076 – 0.067) P = 0.907	-0.025 (-0.097 – 0.046) P = 0.485		0.037 (-0.026 – 0.101) P = 0.248		0.002 (-0.071 – 0.074) P = 0.966
Fasting Insulin	Model 1	0.017 (-0.056 – 0.090) P = 0.647	0.010 (-0.063 – 0.083) P = 0.782	-	-0.018 (-0.092 – 0.056) P = 0.637	-	0.024 (-0.059 – 0.107) P = 0.575
	Model 2	0.020 (-0.054 – 0.094) P = 0.589	0.010 (-0.066 – 0.085) P = 0.798	0.007 (-0.068 – 0.083) P = 0.850	-0.016 (-0.091 – 0.059) P = 0.671	-0.020 (-0.096 – 0.056) P = 0.607	0.027 (-0.057 – 0.111) P = 0.525
	Model 2 (Sens.)[c]	0.009 (-0.079 – 0.096) P = 0.848	0.016 (-0.071 – 0.103) P = 0.719		-0.021 (-0.100 – 0.058) P = 0.597		0.022 (-0.066 – 0.111) P = 0.621
QUICKI	Model 1	-0.012 (-0.085 – 0.060) P = 0.738	-0.008 (-0.080 – 0.065) P = 0.836	-	0.016 (-0.057 – 0.090) P = 0.667	-	-0.036 (-0.118 – 0.046) P = 0.391
	Model 2	-0.015 (-0.088 – 0.059) P = 0.690	-0.009 (-0.084 – 0.066) P = 0.811	-0.006 (-0.081 – 0.069) P = 0.874	0.012 (-0.063 – 0.087) P = 0.754	0.015 (-0.060 – 0.090) P = 0.693	-0.040 (-0.124 – 0.044) P = 0.348

**Model 2 (Sens.)[c]**	-0.010<br>(-0.097 – 0.076)<br>P = 0.812	-0.027<br>(-0.114 – 0.059)<br>P = 0.533	-	0.012<br>(-0.066 – 0.091)<br>P = 0.754	-	-0.041<br>(-0.128 – 0.046)<br>P = 0.356

All results are from multivariate linear regression models. Adjusted for baseline values of respective glycemic traits, age, waist circumference, height, triglycerides, total cholesterol/HDL ratio), hypertension, statin use (model 1), smoking, alcohol consumption, physical activity, CRP, eGFR, TSH, and parental diabetes history (model 2). Effect estimates with 95% CIs were calculated for a one sex-specific SD increase on the log scale of progestogen and estrogen levels, respectively. Significant results are printed in bold. Abbreviations: 17-OHP: 17α-hydroxyprogesterone, CRP: C-reactive protein, eGFR: Estimated glomerular filtration rate, SHBG: Sex hormone-binding globulin, TSH: Thyroid-stimulating Hormone.

[a] *Models were additionally adjusted for albumin.*

[b] *Models were additionally adjusted for SHBG.*

[c] *Sensitivity analyses: Perimenopausal women excluded (n = outcome specific).*

Lau LHY, *et al. BMJ Open Diab Res Care* 2021; 9:e001951. doi: 10.1136/bmjdrc-2020-001951
