# Supplementary material for: Cross-sectional and prospective relationships of endogenous progestogens and estrogens with glucose metabolism in men and women: a KORA F4/FF4 Study
Source: BMJ Open Diabetes Res Care. 2021 Feb 11;9(1):e001951. doi: 10.1136/bmjdrc-2020-001951 (PMC7880095; doi:10.1136/bmjdrc-2020-001951)
Supplement: Supplementary data [file bmjdrc-2020-001951supp003.pdf]

Supplementary Figure 3 – Prospective associations of endogenous progestogens and estrogens with T2D-related traits in men and women of the KORA F4/FF4 cohort*.

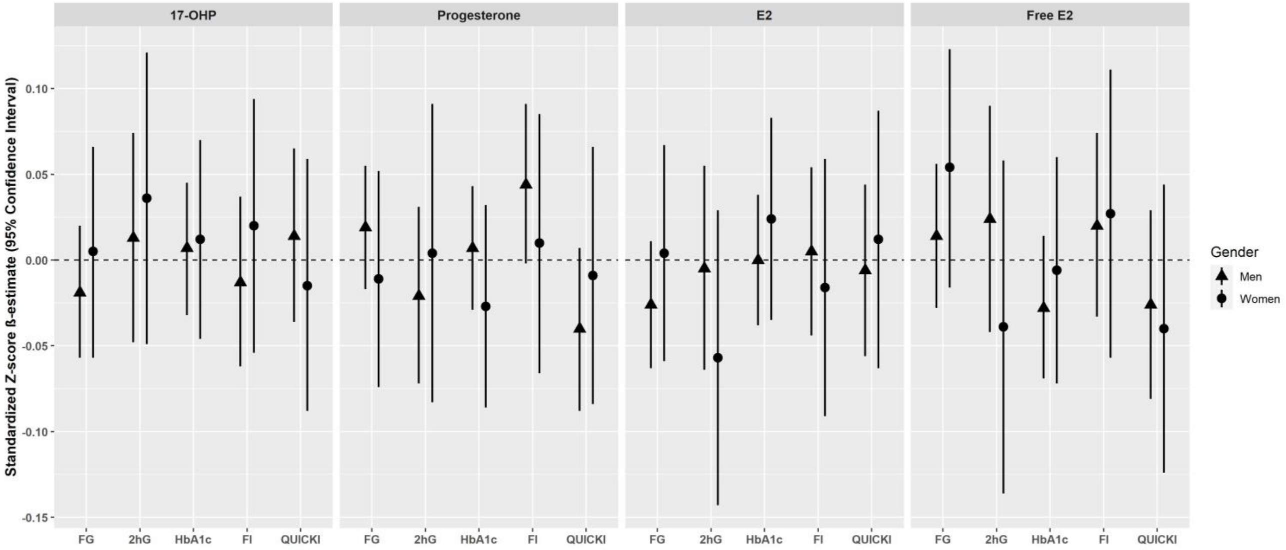

*Results are expressed as the change in one log-unit of the continuous outcome (standardized Z-score β-estimate with 95% CI) per one sex-specific SD increase in the respective progestogens and estrogens. Adjusted for baseline values of respective glycemic traits, waist circumference, height, triglycerides, total cholesterol/HDL-cholesterol ratio, hypertension, statin use, smoking status, alcohol consumption, physical activity, CRP, eGFR, TSH, and parental history of diabetes (model 2). FG: Fasting glucose; 2hG: 2h-glucose; HbA_{1c}: Glycated hemoglobin; FI: Fasting insulin; QUICKI: Quantitative Insulin Sensitivity Check Index, eGFR: estimated glomerular filtration rate, TSH: Thyroid-stimulating hormone. *Men and peri-/postmenopausal women who did not take anti-diabetic medication.*
